# Cancer vaccines in the clinic

**DOI:** 10.1002/btm2.10588

**Published:** 2023-10-27

**Authors:** Morgan E. Janes, Alexander P. Gottlieb, Kyung Soo Park, Zongmin Zhao, Samir Mitragotri

**Affiliations:** ^1^ John A. Paulson School of Engineering & Applied Sciences, Harvard University Cambridge Massachusetts USA; ^2^ Wyss Institute for Biologically Inspired Engineering Boston Massachusetts USA; ^3^ Harvard‐MIT Division of Health Sciences and Technology, Massachusetts Institute of Technology Cambridge Massachusetts USA; ^4^ Department of Pharmaceutical Sciences College of Pharmacy, University of Illinois Chicago Chicago Illinois USA; ^5^ University of Illinois Cancer Center Chicago Illinois USA

**Keywords:** cancer vaccine, clinical trials, dendritic cell, immunotherapy, mRNA, neoantigen, vaccine

## Abstract

Vaccines are an important tool in the rapidly evolving repertoire of immunotherapies in oncology. Although cancer vaccines have been investigated for over 30 years, very few have achieved meaningful clinical success. However, recent advances in areas such antigen identification, formulation development and manufacturing, combination therapy regimens, and indication and patient selection hold promise to reinvigorate the field. Here, we provide a timely update on the clinical status of cancer vaccines. We identify and critically analyze 360 active trials of cancer vaccines according to delivery vehicle, antigen type, indication, and other metrics, as well as highlight eight globally approved products. Finally, we discuss current limitations and future applications for clinical translation of cancer vaccines.

**Translational Impact Statement**

Cancer vaccines have the potential to orchestrate adaptive immune responses to eliminate tumors. Although these vaccines have been the subject of intense clinical investigation for decades, they have yet to become a hallmark treatment in oncology. In this review, we provide a snapshot of this highly active and evolving field via an in‐depth analysis of 360 active clinical trials to illustrate the latest advances and trends in the field. In addition, we discuss longstanding challenges with clinical translation and illuminate novel clinical and preclinical approaches that may help overcome these barriers.

## 1 INTRODUCTION

Vaccines are a critically important public health intervention that have yet to achieve landmark clinical efficacy in oncology.^1^, ^2^ Vaccination holds promise as a treatment modality due to the aberrant overexpression or unique expression of certain antigens on tumor cells. Accordingly, the purpose of a cancer vaccine is to educate a patient's immune system to recognize and attack malignant cells. The success of such vaccines necessitates highly regulated cooperation from many arms of the immune system, including antigen‐presenting cells (APCs), helper and cytotoxic T cells, natural killer (NK) cells, and tumor‐resident myeloid cells. The complex spatiotemporal factors governing such interactions, and the many mechanisms of local and systemic immune suppression and evasion, have long hindered clinical development of cancer vaccines.^3^, ^4^ Their journey has been challenging and wrought with many high‐profile failures. However, an improved understanding of factors such as antigen immunogenicity, immune cell exhaustion, and combination therapy selection holds promise for the application of cancer vaccines in effective therapeutic regimens. Here, we provide an update on the status of cancer vaccines on the market and in the clinic, highlighting 360 active clinical trials (as of July 2022) and eight globally approved products. We dissect the current space according to the materials used to deliver the antigen, antigen types, indications, phases, combination approaches, and other relevant metrics. We then discuss the challenges associated with clinical translation of cancer vaccines, as well as novel preclinical and clinical approaches to circumvent these challenges.

## 2 BACKGROUND AND VACCINE CATEGORIES

**FIGURE 1:**
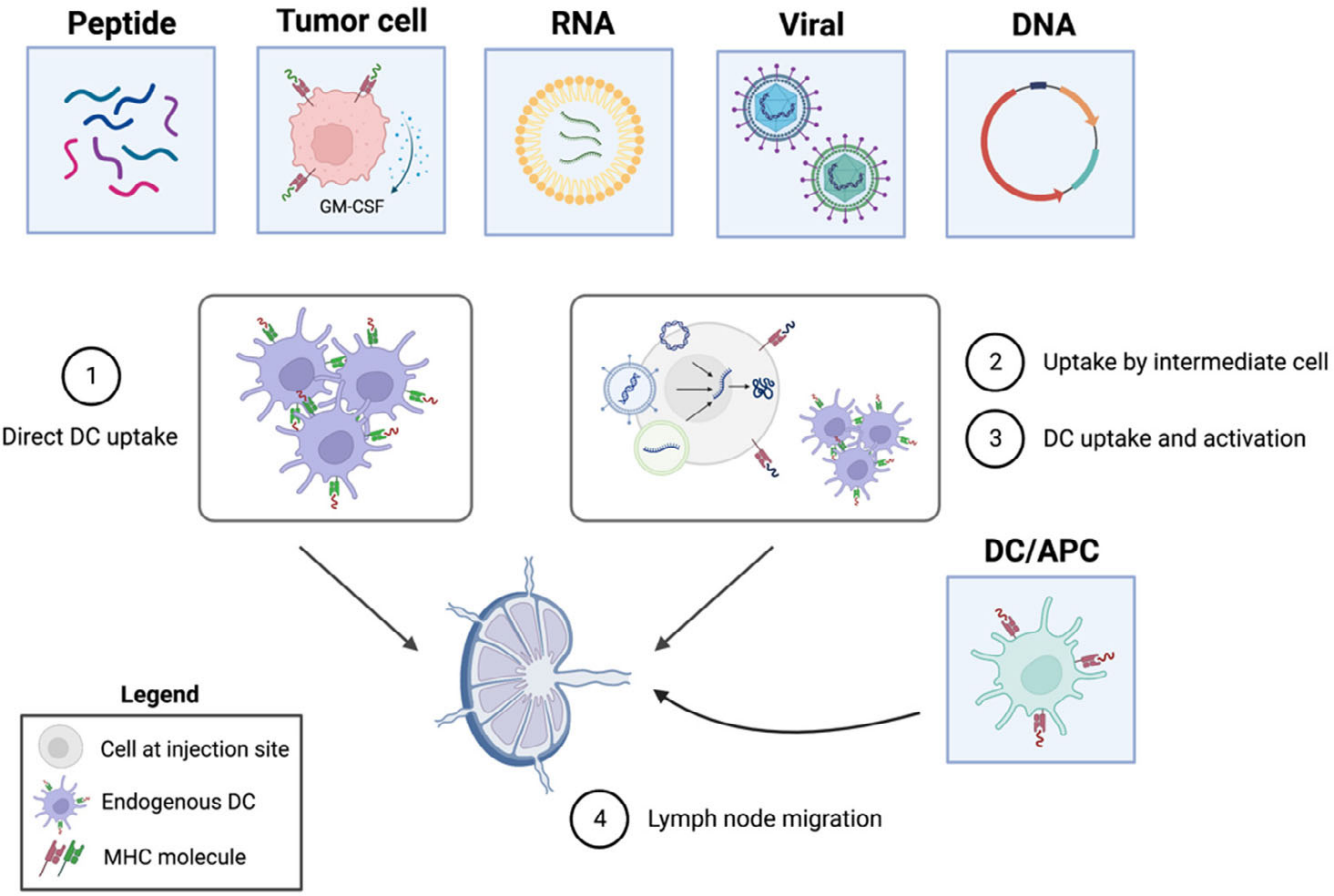
Categories of cancer vaccines and associated mechanisms of action. Peptide, tumor cell, RNA, viral, DNA, and DC/APC vaccines comprise the major categories analyzed. While DC vaccines may directly prime a T cell response, the other constructs all have the potential for direct DC uptake and presentation of antigen (1). Alternatively, non‐cellular constructs may be taken up by cells at the injection site (2), which in turn provides a source of antigen to endogenous DCs (3). Activated DCs then migrate to the lymph nodes, where they induce a T cell response (4). APC, antigen‐presenting cell; DC, dendritic cell; GM‐CSF, granulocyte‐macrophage colony‐stimulating factor; MHC, major histocompatibility complex.

Cancer vaccines have been under intense clinical investigation for 40 years with only a select few successes in very narrow contexts. The earliest fruitful efforts to harness the immune system against cancer were carried out in 1891 by William Coley, who injected live and heat‐killed bacteria into bone and soft tissue sarcomas and observed tumor shrinkage in some patients.^5^ Over 80 years later, intravesical administration of tuberculosis vaccine Bacillus Calmette‐Guérin (BCG) demonstrated efficacy against non‐muscle‐invasive bladder cancer through local immune activation that impairs tumor cell survival and proliferation.^6^ BCG therapy remains standard in bladder cancer and represents the first modern approved cancer immunotherapy. The early bacterial approaches resulted in localized immune activation against foreign antigens, spurring the development of anti‐tumor immune responses. Today, most vaccines under clinical investigation involve the delivery of tumor antigens in combination with an adjuvant or other costimulatory factor. In this review, we focus on six major categories of cancer vaccines including peptides, RNA, DNA, tumor cells, and viral vaccines, and APC vaccines, most of which consist of dendritic cells (DCs) (Figure 1).

Some of the earliest cancer vaccines used tumor cells; in 1993, promising preclinical results were reported from lethally irradiated tumor cells genetically engineered to secrete granulocyte‐macrophage colony‐stimulating factor (GM‐CSF).^7^ In this vaccination scheme, tumor cells act as a source of the entire array of immunogenic antigens, while their irradiation prior to injection renders them replication‐incompetent and enhances their immunogenicity. GM‐CSF recruits monocytes and DCs to the injection site and contributes to their survival and maturation, such that mature, antigen‐bearing DCs may return to the lymph nodes to initiate an immune response.^8^, ^9^ This tumor cell‐based approach, termed GVAX, was translated to human trials in a wide range of indications, with autologous and allogeneic cells used for personalized and off‐the‐shelf vaccines, respectively. Despite promising preclinical results and immune responses observed in many patients, two Phase 3 trials (VITAL‐1 and VITAL‐2) were halted in 2008 for futility.^10^ While GVAX remains under clinical investigation in some Phase 1 and 2 trials, particularly to evaluate the therapy in combination with immune checkpoint blockade (ICB) or chemotherapy, the most intensive development efforts have since shifted to other vaccination approaches. A detailed review of the clinical history of GVAX may be found elsewhere.^11^

Rather than delivering a source of tumor antigens and relying on endogenous APCs to initiate the immune response, DC vaccines instead aim to supply fully differentiated, mature DCs which theoretically possess all signals necessary to induce an anti‐tumor immune response, including antigen presentation, costimulatory signals, and cytokine production.^12^, ^13^, ^14^ Typically, monocytes are harvested from the patient via leukapheresis and cultured ex vivo with GM‐CSF for at least 1 week to yield differentiated monocyte‐derived DCs (moDCs). At this point, the cells are loaded with tumor antigens and matured with an activation cocktail before reinfusion, usually via the subcutaneous (SC) or intradermal (ID) route. While this approach is appealing because it can overcome endogenous DC dysfunction that is often observed in cancer, it bears major logistical hurdles and a high cost. Autologous DC vaccines have undergone rigorous clinical investigation, beginning in the 1990s with the identification of tumor‐associated antigens suitable for vaccination.^15^ A significant majority of past DC vaccine trials employed moDC, and modest research efforts were devoted to identifying optimal protocols for ex vivo activation of these cells.^16^ Modern protocols precondition moDC with activation cocktails of cytokines and Toll‐like receptor (TLR) agonists to elicit maximal expression of costimulatory receptors and production of key cytokines while preserving cell viability and migratory capacity. Various protocols for culturing, antigen loading, and maturing DC vaccines have been reviewed in depth elsewhere.^17^

Clinical responses to DC vaccines have been largely disappointing, with response rates rarely exceeding 15%.^18^ Multiple Phase 3 trials have failed to produce desired results, with only a single APC‐based approach (Sipuleucel‐T) showing sufficient efficacy to support regulatory approval. Many DC vaccines have succeeded in eliciting measurable anti‐tumor T cell responses, but these responses have been insufficient to consistently yield clinical benefit. These disappointments have been attributed to a variety of factors, including duration and stability of antigen presentation, suboptimal characteristics or exhaustion of anti‐tumor CD8 T cells, insufficient priming of Th1‐polarized CD4 T cells, and failure to repolarize the suppressive tumor microenvironment (TME) to enable T cell function.^19^, ^20^ Perhaps the primary limitation is the inability to differentiate and expand the most relevant DC subset for the activation of CD8 T cells, conventional type I DCs (cDC1), in an ex vivo setting. While moDC are easily obtained in large quantities, they are comparatively deficient in their cross‐presentation and T cell stimulatory capacities.^21^, ^22^ The past decades of clinical research have produced a wealth of insights into the challenges facing DC vaccination, and ongoing preclinical and clinical studies aim to address the deficiencies of previous approaches. Chief among these strategies are combination with ICB, optimization of antigen loading methods, novel protocols to culture specialized DC, and inclusion of CD4 epitopes.^23^, ^24^, ^25^ As with the entire cancer vaccine field, recent advances in gene sequencing and immunogenic neoantigen identification promise to enhance DC vaccines, and cutting edge approaches have focused on loading DCs with patient‐specific neoantigens using messenger RNA (mRNA) transfection.^17^ While hard‐won clinical insights promise to bring ongoing and future approaches closer to their clinical potential, the time and resources required to generate these autologous cellular vaccines remain a barrier.

Compared with DC vaccines, peptide‐based cancer vaccines are simpler and less expensive to design, manufacture, and administer. In this vaccination scheme, peptides encoding tumor antigens are injected, typically into the subcutaneous space or dermis, to supply APCs with relevant antigens. Paramount to the efficacy of peptide‐based vaccines is proper adjuvanting of the vaccine, as peptides are typically weakly immunogenic. Adjuvants are co‐administered with antigen peptides to mature local APCs, often but not exclusively through activation of pattern recognition receptors (PRRs) designed for pathogenic surveillance. Common vaccine adjuvants include oil emulsions like Montanide ISA‐51 or double‐stranded oligonucleotides like poly‐ICLC; cancer vaccine adjuvants are reviewed in depth in other sources.^26^ Peptide‐based vaccines are often mixed with GM‐CSF immediately prior to administration, as the cytokine can help to recruit and mature DCs. Extensive preclinical research efforts have gone into the design of particle‐ or material‐based systems to coordinate spatial and temporal control over exposure to antigen and adjuvant; these approaches are reviewed elsewhere.^27^ Early trials of peptide‐based vaccines prioritized the administration of major histocompatibility complex (MHC) class I‐restricted minimal epitopes, 8–9 amino acid peptides that can form peptide–MHC complexes for the activation of T cells without the need for uptake and processing by antigen‐presenting cells. The field has since moved away from minimal epitopes towards synthetic long peptides (SLPs), as the presentation of minimal epitopes on non‐APC bystanders likely contributes to T cell anergy.^28^ Peptide vaccines are among the most actively studied in Phase 3 trials, but the results have been almost uniformly disappointing.^29^, ^30^, ^31^, ^32^, ^33^

DNA‐based cancer vaccines deliver plasmids containing genetic material encoding tumor antigens, relying on endogenous cells to take up the construct and express the encoded antigens. These vaccines are somewhat less dependent on adjuvants than peptide‐based vaccines, as the injection of foreign genetic material is inherently immunogenic, but many trials also co‐administer an adjuvant.^34^ Plasmids can also be engineered to support expression of co‐stimulatory factors such as cytokines and chemokines. As the body's own cells manufacture tumor antigens, there are possible benefits to antigen stability over peptide‐based vaccines, and DNA is typically very stable under a variety of storage conditions. Owing to the need for intracellular delivery, DNA uptake and expression have historically been barriers to the efficacy of these vaccines.^35^ More recent trials employ electroporation following intramuscular vaccination; electroporation temporarily disrupts the lipid bilayer of endogenous cells to allow uptake of surrounding materials – in this case, cytosolic delivery of DNA for its expression. DNA vaccines have been actively explored in the clinic in the past, and with the advent of clinical electroporation, this modality is attracting renewed interest.^36^

RNA vaccines are a cutting‐edge cancer vaccine approach that also involves the delivery of oligonucleotides encoding the desired tumor antigens. mRNA vaccines typically deliver a synthetic, modified mRNA encoding multiple tumor antigens in a lipid‐based delivery vehicle that protects the mRNA from degradation and facilitates its cellular uptake and expression. These cationic lipids likely also act as adjuvants and contribute to vaccine immunogenicity.^37^, ^38^ In a recent major advance, the composition of such carriers may be tuned to optimize delivery to DCs via the intravenous (IV) route.^39^ The clinical efficacy of these vaccines is being evaluated for the first time for therapeutic efficacy against cancer and infectious disease prophylaxis in ongoing trials. These vaccines typically target patient‐specific neoantigens, as a great deal of logistical effort and expense is required for manufacturing regardless of antigen identity. mRNA vaccines have recently demonstrated promising clinical results, generating great excitement as an emerging standard in the field.^40^

Viral vaccines similarly aim to deliver genetic material into endogenous cells to achieve antigen expression. In viral vaccination schemes, an engineered, infectious but often replication‐incompetent virus is administered to infect host cells. Host cells express the encoded antigens and are rendered immunogenic by virtue of their infection, supplying vaccine adjuvanticity.^41^ An inherent weakness of this strategy is the development of a humoral response against the vaccine vector itself, which limits repetitive dosing with vector‐neutralizing antibodies. Therefore, many viral vaccination approaches employ a heterologous approach, in which one virus type may be used for priming and another for boosting.^42^ These vaccines have been under clinical investigation for decades, but they have not yet demonstrated efficacy in a Phase 3 trial. Efforts to develop these cancer vaccines are ongoing.

## 3 APPROVED PRODUCTS

We investigated the global landscape of approved cancer vaccines and identified eight products that have been used commercially.^43^ However, the clinical results for these products have on the whole been disappointing. Sipuleucel‐T (Provenge®) is the sole cancer vaccine approved for clinical use in the United States. In a Phase 3 trial (IMPACT), Sipuleucel‐T prolonged overall survival by 4.1 months in patients with metastatic castration‐resistant prostate cancer.^44^ The therapy consists of autologous peripheral blood mononuclear cells (PBMCs), which include various APCs, pulsed ex vivo with a fusion protein of GM‐CSF and prostatic acid phosphatase, a tumor‐associated antigen. Notably, the PBMCs are not differentiated ex vivo into a uniform dendritic cell product. The survival benefit observed in the IMPACT trial was sufficient to win Food and Drug Administration (FDA) approval, however, there was no improvement in progression‐free survival or any clear reduction in tumor volume, prompting concerns that the observed benefit was attributable to shorter‐than‐expected survival in the study control arm.^45^ With uncertainty surrounding the therapy's modest clinical benefit, significant logistical challenges, and high price with doubtful cost‐effectiveness, the therapy failed to achieve widespread uptake or clinical impact and is poorly available today.^46^, ^47^

A total of seven cancer vaccines have been approved for commercial use outside the United States, largely without Phase 3 trial data. Hybricell is a DC‐tumor cell fusion approved in Brazil in 2005; a Phase 3 trial was never conducted. Creavax‐RCC is a DC vaccine that was “tentatively approved” to treat renal cell carcinoma (RCC) by the Korean FDA in 2007, awaiting results from a Phase 3 trial approved that year.^48^ No further data could be identified to support the efficacy of this product. APCEDEN is another DC vaccine that was approved to treat non‐small cell lung cancer (NSCLC), colorectal cancer (CRC), ovarian, and prostate cancer by the Indian FDA in 2017.^49^ This approval was based on a 2014 single‐arm, open label study and a 2017 retrospective analysis; these data are unlikely to support further regulatory approvals.^50^, ^51^ Oncophage is an autologous heat shock protein‐peptide vaccine approved in Russia for RCC in 2008 based on a Phase 3 trial; however, following concerns over data integrity, the company withdrew its European Medicines Agency (EMA) application in 2009.^52^, ^53^ A later US‐based Phase 3 study identified no clinical benefit.^54^

Two cancer vaccines have been approved in Switzerland: M‐vax and DCvax‐Brain, a modified tumor cell vaccine in melanoma and DC vaccine in glioblastoma (GBM), respectively. M‐vax has not shown efficacy in a Phase 3 trial, and is no longer available.^55^ DCvax‐Brain, alternately referred to as DCvax‐L, consists of autologous DCs loaded with autologous tumor lysate and has been under clinical investigation for nearly two decades. DCvax‐Brain earned a hospital approval in Germany in 2014, allowing its administration with reimbursement for 5 years.^56^ The developing company, Northwest Biotherapeutics, has recently released results from a long‐running Phase 3 trial reporting a 3–5 month survival benefit; however, substantial concerns surround the externally controlled trial, and regulatory approval remain to be seen.^57^ Finally, CIMAVAX‐EGF is a Cuban‐developed epidermal growth factor (EGF)‐bacterial fusion protein vaccine adjuvanted with Montanide ISA‐51 for the treatment of NSCLC. A significant survival benefit was identified in a subpopulation of a Cuban Phase 3 trial in 2016, supporting earlier approvals as a switch maintenance therapy in Cuba, Peru, Paraguay, Colombia, and Bosnia.^58^, ^59^ The vaccine is now undergoing early‐stage, US‐based clinical trials, (NCT04298606, NCT02955290).

## 4 CLINICAL LANDSCAPE AND METHODOLOGY

A total of 360 cancer vaccine trials were analyzed in the present study. We identified trials on clinicaltrials.gov using the following key words in the “Other terms” category: “cancer vaccine” (automatically searched for “tumor vaccine,” “cancer treatment vaccine,” “antineoplastic vaccine,” and “neoplasm vaccine”), “dendritic cell vaccine” (automatically searched for “antigen presenting cell”), “in situ vaccine,” “monocyte vaccine” (automatically searched for “monocytic”), and “PBMC vaccine” (automatically searched for “peripheral blood mononuclear cells”). In the recruitment status category, we selected trials designated as “not yet recruiting,” “recruiting,” “enrolling by invitation,” and “active/not recruiting.” The data reflect the trial space as of July 2022. From the initial pool of trials, we eliminated irrelevant listings, including: (i) those with indications other than cancer, (ii) those that had been active for 10+ years without release of expected results, and (iii) those that did not fit the definition of a vaccine. For the purpose of this study, cancer vaccines were defined as containing or encoding tumor antigens. The singular exception was in situ vaccines, which were explicitly searched and were required to be designated as such in the trial listing. Vaccines designed to induce a response against non‐cancer antigens, such as the influenza vaccine, were excluded unless combined with a tumor‐specific vaccine. Prophylactic vaccines were included and analyzed separately from the major subcategories. Vaccines designed to prevent recurrence of cancers in remission were not designated as prophylactic and were included in the general analysis. Nonavalent, previously approved human papillomavirus (HPV)‐targeted vaccines for the prevention of HPV‐associated cancers (i.e., Gardasil) were excluded entirely, however, novel prophylactic HPV vaccines were included. Due to inconsistencies in terminology used for clinical reporting, it is unlikely that all cancer vaccine trials were captured in this analysis.

**FIGURE 2:**
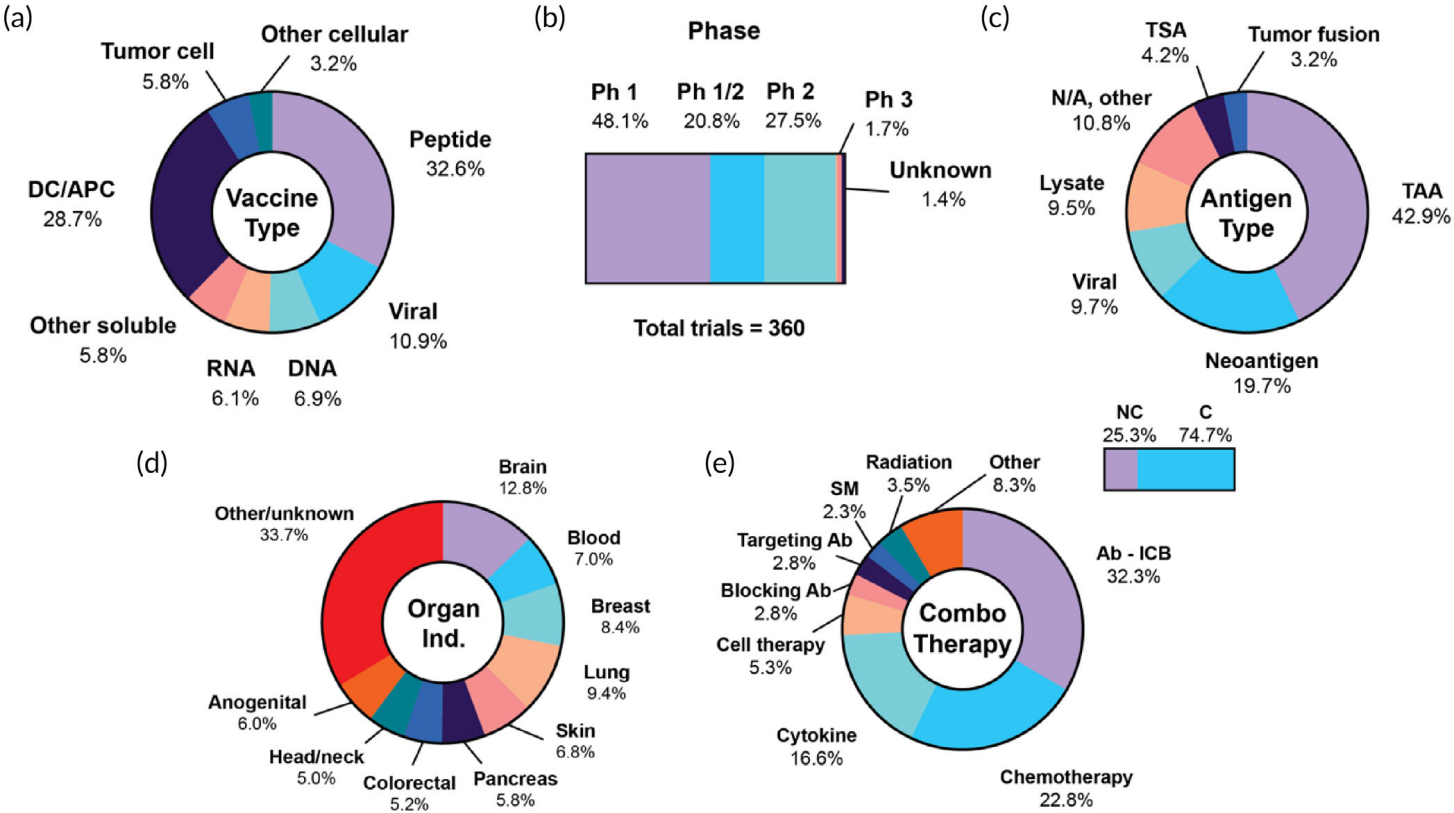
Summary of current landscape of cancer vaccine clinical trials. (a) Overview of all captured interventions by type, (b) phases of 360 clinical trials, (c) landscape of antigen classifications, (d) most common indications segmented by organ/anatomic site, (e) percentage of trials using a combination therapy (top right bar), and combination therapies by category, among trials using a combination. Ab, antibody; APC, antigen‐presenting cell; C, combination; DC, dendritic cell; ICB, immune checkpoint blockade; N/A, not applicable; NC, no combination; Ph, phase; SM, small molecule; TAA, tumor‐associated antigen; TSA, tumor‐specific antigen.

The trials were segmented according to the use of soluble or cellular materials to deliver the antigen(s). The major subcategories among the soluble vaccines include peptides, RNA, DNA, viral and heterologous, and other (Figure 2). Because the vast majority of heterologous approaches employed at least one viral element, these categories were grouped together for downstream analysis. These trials are not counted twice in the subsection analyses of other categories such as RNA and DNA, unless explicitly stated. The major subcategories among the cellular vaccines are DC/PBMC, tumor cell, and other. The DC subcategory encompasses DC, PBMC, APC, and monocyte‐based vaccines. Because various trials employ more than one intervention, Figure 2a is represented as the total number of interventions identified by category.

Of the 360 trials analyzed, we identified 377 distinct vaccine interventions on a per‐trial basis, comprising 235 soluble (62.3%) and 142 cellular (37.7%) vaccines. Peptide vaccines comprise more than half the soluble vaccine space (123/235, or 52.3%) and were the most dominant singular category overall (123/377, or 32.6%). DC vaccines are the second most frequently investigated, and comprise the vast majority of the cellular vaccine space (108/142, 76.1%) and a large fraction of the total cancer vaccine space (28.7%). Viral (10.9%), DNA (6.9%), RNA (6.1%), and tumor cell vaccines (5.8%) followed DC vaccines. The majority of trials are at Phase 1 or 1/2 (68.9%), followed by Phase 2 (27.5%), and Phase 2/3 and 3 (7 trials, 1.7%), reflecting both the difficulty of clinical advancement in the space and wealth of new products that continue to enter clinical trials (Figure 2b).

When broken down by the type of antigen, the most represented category is tumor‐associated antigens (TAAs) (163/380, 42.9%), antigens that are overexpressed on tumor cells. Vaccines also target neoantigens and tumor‐specific antigens (TSAs) (75/380 and 16/380, 23.9% combined) (Figure 2c). These antigens are exclusively expressed on tumor cells and absent from healthy cells. In this review, TSA denotes a singular defined vaccine product targeting a “shared” mutation. For TSA vaccines, patients are screened to determine if they possess the mutation in question and are eligible for the therapy. “Neoantigen” denotes a vaccine consisting of patient‐specific antigens that are absent from healthy cells. For neoantigen vaccines, the patient's individual tumor is sequenced to identify these patient‐specific antigens and produce a personalized product. Other antigen types include viral antigens (9.7%), indicated for virus‐associated cancers, and antigens derived from tumor lysate (9.5%). Tumor fusion vaccines are DC vaccines fused with tumor cells prior to infusion (3.2%). Trials where the type was not applicable (N/A, Figure 2c) refer to tumor cell and in situ vaccine trials.

The most commonly targeted organ is the brain, followed by cancers of the blood, breast, lung, skin, pancreas, and prostate (Figure 2d). Of the 360 trials, 269 (74.7%) employ a combination approach (Figure 2e). Among the combination strategies, the most common is ICB (32.3%), followed by chemotherapy (22.8%), cytokines (16.6%), and cell therapies (5.3%). To analyze these data, we counted the number of times a combination type was listed and calculated its frequency as a percentage of the total number of combination interventions in that category. Therefore, the data represent the prevalence of the combination type among all combination interventions, not the fraction of trials that use that type of therapy. Many trials use more than one approach together or in different arms of the trial, which is reflected in the large total number of identified interventions (419) compared with the number of combination trials (269).

## 5 CURRENT CLINICAL TRIALS

### 5.1 Peptide vaccines

**FIGURE 3:**
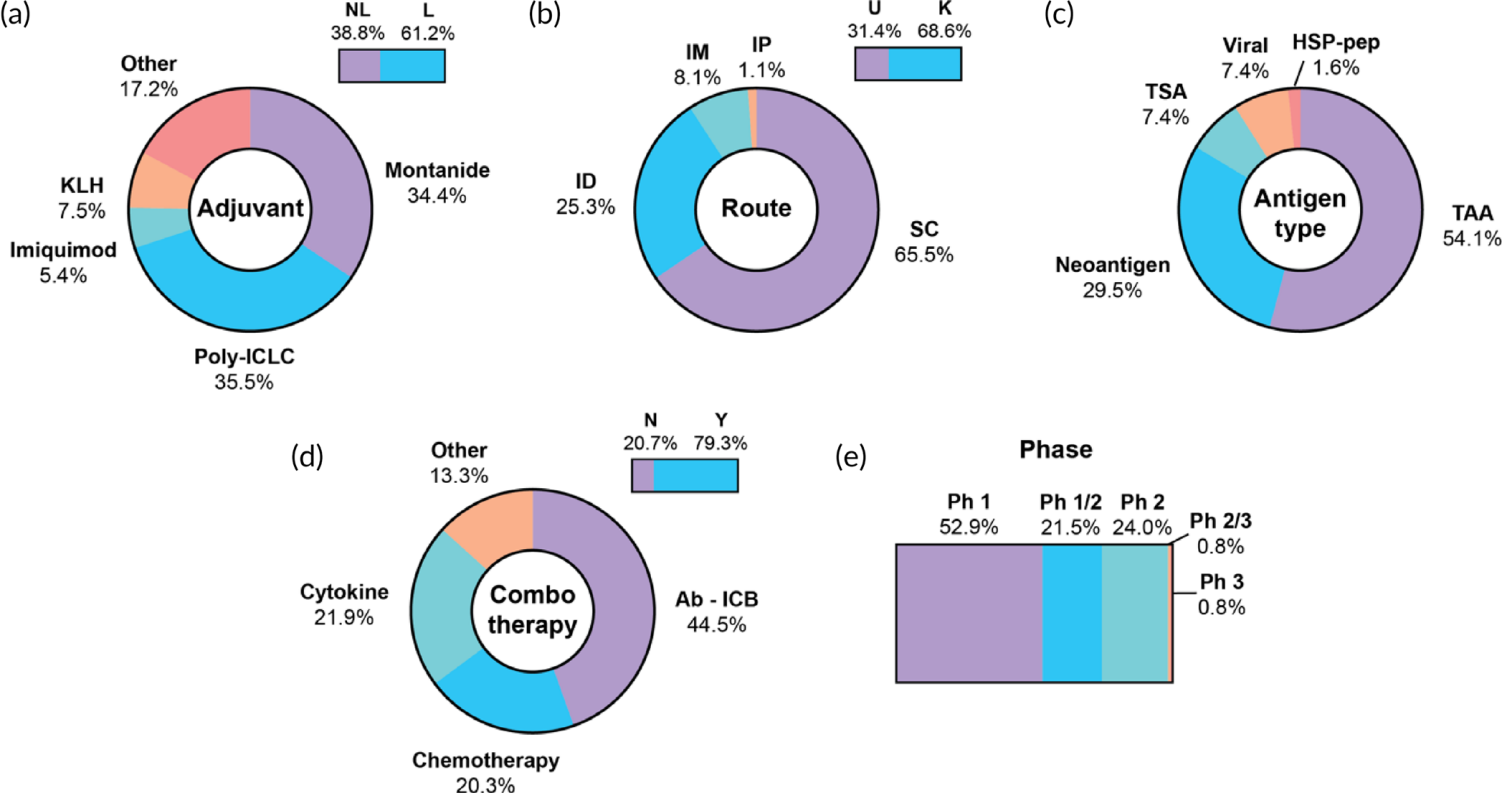
Features of clinical trials employing peptides as antigen material. (a) Percentage of trials with a listed adjuvant (top right bar) and adjuvant types among those trials, (b) percentage of trials with a listed route of administration (top right bar) and routes among those trials, (c) antigen classification, (d) percentage of trials using a combination therapy (top right bar), and combination therapies by category, among trials using a combination (e) breakdown of trial phases. Ab‐ICB, antibody‐immune checkpoint blockade; HSP‐pep, heat shock protein‐peptide; ID, intradermal; IM, intramuscular; IP, intraperitoneal; K, known; KLH, keyhole limpet hemocyanin; L, listed; N, no; NL, not listed; Ph, phase; SC, subcutaneous; TAA, tumor‐associated antigen; TSA, tumor‐specific antigen; U, unknown; Y, yes.

Peptide‐based cancer vaccines are appealing for their relatively low cost, straightforward and expedient manufacturing, and potential for personalization. These vaccines are employed in 123/360 (34.2%) ongoing clinical trials, the most of any vaccine modality (Figure 3). A representative selection of trials can be found in Table 1. Peptide‐based vaccines are used to treat cancers occurring in a wide range of target sites, chief among them brain (15%), lung (12%), breast (10%), and skin (8%) (Table S1). As peptide vaccines largely aim to load endogenous APCs with tumor antigens for the initiation of cellular immune responses, proper maturation of APCs is paramount to vaccine efficacy.^26^ Curiously, 38.8% of peptide‐based vaccine trials did not explicitly list an adjuvant. Among trials with listed adjuvants, poly‐ICLC (35.5%) and Montanide (32.3%), were the most common by far. Both adjuvants are inexpensive and generally well‐tolerated (Figure 3a).^60^ Among trials with a listed administration route, vaccines are most commonly administered via subcutaneous (SC) (65.5%) and intradermal (ID) (25.3%) routes (Figure 3b). Peptides injected SC are expected to drain to lymph nodes for uptake and antigen processing; those injected ID may drain to the lymph nodes or be taken up and processed by dermal APCs.

**TABLE 1:** Examples of current clinical trials for peptide cancer vaccines.

NCT	Phase	Sponsor/collaborator	Product name	Indication	Antigen type	Antigens	Adjuvants	Route	Combination therapy	Specific combination	Extra details	Other trials
NCT02358187	2	Ian F Pollock	GAA/TT	Pediatric low‐grade glioma	TAA	GAA	PolyICLC	SC	N/A	N/A	Tetanus toxoid conjugate	N/A
NCT04930783	1	Dana Farber Cancer Institute, Celldex Therapeutics	NeoVax	Advanced melanoma	Neoantigen	N/A	PolyICLC	SC	Antibody‐ICB, cytokine	Nivolumab, CDX‐301	Long neoantigen peptides	NCT03929029, NCT02287428, NCT03361852, NCT03219450, NCT02950766, NCT04024878
NCT04580771	2	MD Anderson Cancer Center	PDS0101	Cervical cancer	Viral	HPV E6, E7	N/A	SC	Chemotherapy	Cisplatin	Delivered via liposome	N/A
NCT04382664	2	Ultimovacs ASA	UV1	Melanoma	TAA	hTERT	N/A	ID	Antibody‐ICB, cytokine	Nivolumab, ipilimumab, GM‐CSF	N/A	NCT04300244, NCT04046445, NCT03538314, NCT05075122, NCT01784913, NCT01789099
NCT02960230	1/2	Sabine Mueller	H3.3K27M	Diffuse intrinsic pontine glioma	TSA	H3.3K27M	Montanide ISA 51 VG, tetanus toxoid, polyICLC	SC	Antibody‐ICB	Nivolumab	N/A	N/A
NCT02654587	3	OSE Immunotherapeutics	OSE2101	Non‐small cell lung cancer	TAA	CEA, HER2, MAGE2, MAGE3, P53, PADRE	N/A	SC	N/A	N/A	N/A	NCT04713514

Abbreviations: HPV, human papillomavirus; ICB, immune checkpoint blockade; ID, intradermal; TAA, tumor‐associated antigen, TSA, tumor‐specific antigen, SC, subcutaneous.

A majority (54.1%) of peptide‐based vaccines target tumor‐associated antigens (TAAs) (Figure 3c). Among the 30 identified Phase 2 trials, 24 (80%) target TAAs, with hTERT (23%) and HER2 (13%) most commonly targeted (Table S2). Neoantigen targets were mentioned 36 times (29.5%); two neoantigen trials are in Phase 2 (7% of all Phase 2 trials), while 34 are in Phase 1 and 1/2 (37% of all Phase 1 and 1/2 trials). This disparity likely reflects relatively recent advances in individualized sequencing, computational antigen prediction, and small‐batch production.^61^, ^62^ The clinical advancement of neoantigen‐targeting peptide‐based vaccines may progress rapidly as these barriers to antigen identification continue to fall.

Interestingly, only 25 trials (20.7%) administer peptide‐based vaccines as monotherapies. Vaccines are most frequently combined with ICB (44.5%), chemotherapy (20.3%), and cytokine therapy (21.9%) (Figure 3d). While ICB and chemotherapy may help to control tumor burden synergistically with vaccination, these statistics also likely reflect the reality that peptide vaccines are rarely sufficiently potent as monotherapies.

The vast majority of peptide vaccine trials are at Phase 1 or 1/2 (74.4%) (Figure 3e). We identified only one peptide‐based trial in Phase 3, carried out in NSCLC patients non‐responsive to ICB (NCT02654587). Tedopi (OSE2101, OSE Immunotherapeutics) is a peptide vaccine targeting five TAA along with CD4 helper epitope PADRE. Overall survival was significantly extended (hazard ratio (HR) = 0.59, *p* = 0.017) in treated patients, motivating a compassionate use authorization in the European Union (EU).^63^ This trial was halted at the onset of the coronavirus disease (COVID‐19) pandemic, and a confirmatory Phase 3 trial is awaited.

### 5.2 RNA vaccines

**FIGURE 4:**
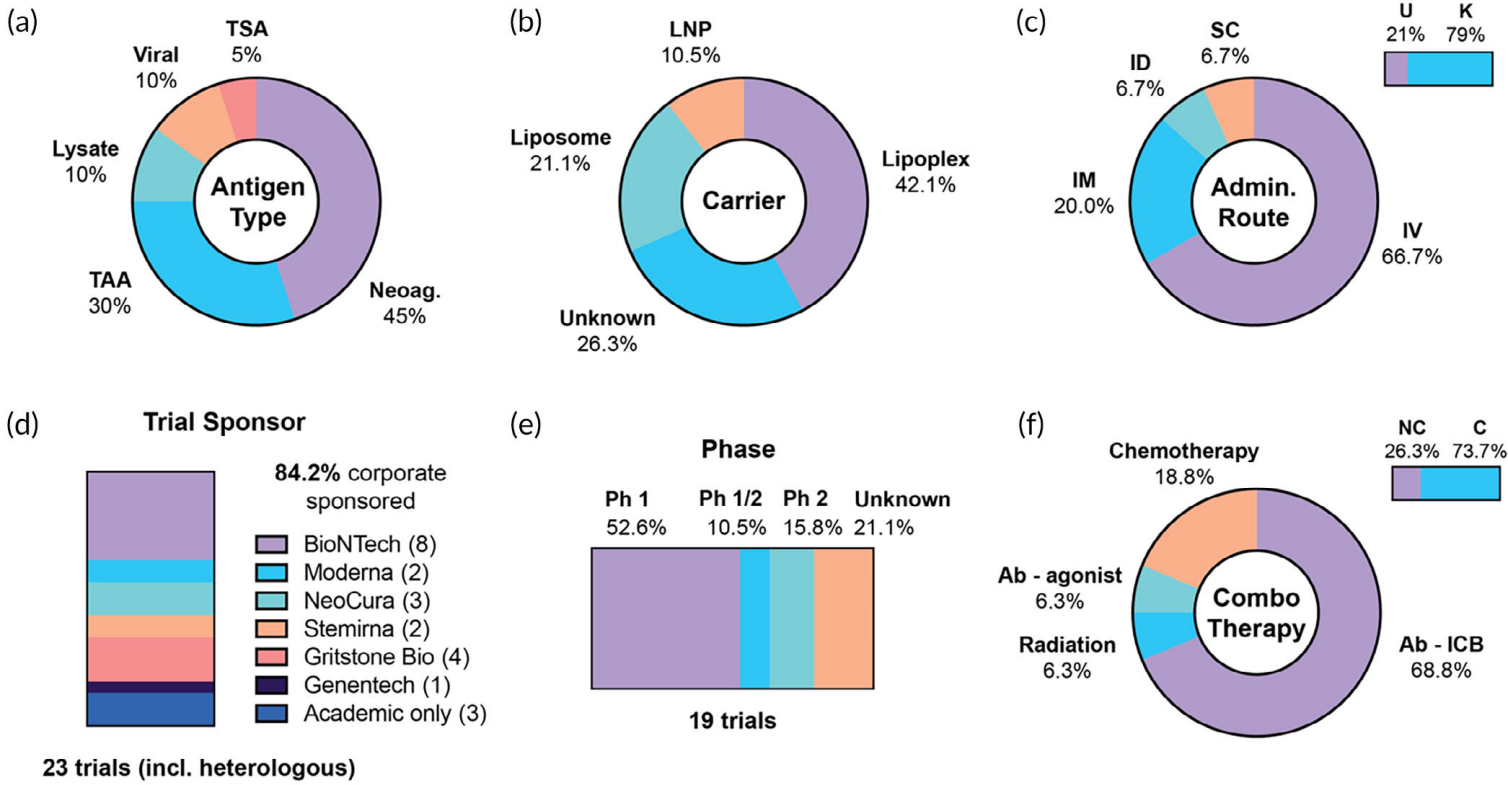
Overview of clinical trials using RNA as antigen material. (a) Antigen classification, (b) type of drug carrier, (c) percentage of trials with a known route of administration (top right bar) and breakdown among trials with a listed route, (d) summary of trial sponsors, including RNA modalities used with viral/heterologous vaccines, (e) breakdown of trial phases, (f) percentage of trials using a combination therapy (top right bar), and combination therapies by category, among trials using a combination. Ab‐agonist, antibody‐agonist; Ab‐ICB, antibody‐immune checkpoint blockade; C, combination; LNP, lipid nanoparticle; Neoag, neoantigen; TAA, tumor‐associated antigen; TSA, tumor‐specific antigen; ID, intradermal; IM, intramuscular; IV, intravenous; K, known; NC, no combination; Ph, phase; SC, subcutaneous; U, unknown.

RNA cancer vaccines are rapidly entering clinical‐stage investigation.^64^, ^65^ The current clinical thrust has been enabled by two critical areas of technical advancement: (i) the development and validation of drug delivery carriers to protect RNA cargo in vivo, and (ii) recent improvements in computational tools to identify immunogenic epitopes, towards the development of effective neoantigen vaccines.^61^, ^66^ We identified 23 trials employing RNA vaccines, comprising 6.4% of the 360 total trials (Figure 4). A representative selection is included in Table 2. This number is expected to increase significantly in the coming years. Four of these trials employ a heterologous prime‐boost approach in combination with a viral vaccine, and they are discussed in more detail in section 5.4.

**TABLE 2:** Examples of current clinical trials for RNA cancer vaccines.

NCT	Phase	Sponsor/collaborator	Product name	Indication	Delivery vehicle	Antigen type	Antigens	Route	Combination therapy	Specific combination	Other trials
NCT03897881	2	Moderna	mRNA‐4157	Melanoma (resected)	Lipid nanoparticle	Neoantigen	N/A	IM	Antibody‐ICB	Pembrolizumab	NCT03313778
NCT04526899	2	BioNTech SE	BNT111	Melanoma (checkpoint refractory/unresectable)	Lipoplex	TAA	NY‐ESO‐1, MAGE‐A3, tyrosinase, TPTE	IV	Antibody‐ICB	Cemiplimab	N/A
NCT03418480	1/2	University of Southampton, BioNTech SE	HARE‐40	Head and neck, cervical, anogenital	Lipoplex	Viral	HPV E6, E7	ID	Antibody‐agonist	Anti‐CD40	N/A
NCT04573140	1	University of Florida	RNA‐LP	Glioblastoma	Liposome (DOTAP)	Lysate (autologous), viral	TTRNA from lysate, pp65‐LAMP	IV	Radiation	N/A	N/A
NCT04534205	2	BioNTech SE	BNT113	Head and neck squamous cell carcinoma	Lipoplex	Viral	HPV E6, E7	IV	Antibody‐ICB	Pembrolizumab	N/A
NCT04382898	1/2	BioNTech SE	BNT112	Prostate cancer (metastatic, castration‐resistant)	Lipoplex	TAA	PAP, PSA, and three undisclosed	IV	Antibody‐ICB	Cemiplimab	N/A

Abbreviations: HPV, human papillomavirus; ICB, immune checkpoint blockade; ID, intradermal; IM, intramuscular; IV, intravenous; PAP, prostatic acid phosphatase; PSA, prostate‐specific antigen; TAA, tumor‐associated antigen; TTRNA, total tumor RNA.

Across the space, RNA vaccines targeting neoantigens are the most common (9/20 antigen types, 45%), followed by TAAs (6/20, 30%) (Figure 4a). One trial contains a TSA target, which is a shared KRAS mutant. Two trials have a viral (HPV) target, while another two consist of total tumor RNA derived from tumor lysate. Three distinct types of lipid‐based carriers were identified, encompassing lipoplexes, liposomes, and lipid nanoparticles (LNPs) (Figure 4b). Their usage is mainly stratified by sponsor, with BioNTech and Stemirna utilizing lipoplexes and Moderna opting for LNPs. Liposomes consist of a lipid bilayer surrounding an aqueous phase with encapsulated cargo, while LNPs contain a lipid core that is often stabilized by surfactants.^67^ Lipoplexes differ slightly in that they consist of a cationic lipid that directly self‐assembles with mRNA.^39^ Injection route is also an important consideration for efficacy of RNA vaccines, and must be considered in the context of the carrier. Among trials that specified a route, the majority of vaccines are injected IV, with others injected via intramuscular (IM) or ID routes (Figure 4c). IM and ID injections have generally been shown to promote more persistent antigen expression.^68^ However, lipoplex vaccines developed by BioNTech demonstrate a tropism for secondary lymphoid organs and bone marrow, which facilitates DC uptake and a strong immune response.^39^ The abundance of trial listings by BioNTech (discussed below) governs the dominance of the IV route in the current landscape.

The indications among the 19 trials are highly varied, and all are used for solid tumors (Table S3). Of the three Phase 2 trials, two are indicated for melanoma (NCT03897881, NCT04526899), and the other for head and neck squamous cell carcinoma (HNSCC) (NCT04534205). Interestingly, 87% (20/23) of all RNA trials (including heterologous approaches with viral vectors) have a listed corporate sponsor or partner, likely owing to the expense of RNA therapeutic development (Figure 4d). BioNTech SE is listed on 8/23 trials, and Moderna is listed on 2/23. Both companies have reached the Phase 2 stage in melanoma. Moderna's lead candidate, mRNA‐4157, is composed of a lipid nanoparticle encapsulating mRNA encoding up to 20 patient‐specific neoantigen sequences.^69^ It is administered IM in combination with the anti‐PD‐1 antibody pembrolizumab in two trials for melanoma (NCT03897881) and multiple solid tumors (NCT03313778). In late 2022, mRNA‐4157 met its primary endpoint in the KEYNOTE trial (NCT03897881), improving recurrence‐free survival in completely resected melanoma compared with checkpoint blockade alone.^40^ This was a critical efficacy milestone for the field that underscores multiple recent technical and logistical improvements, including LNP formulation, neoantigen prediction, optimization of RNA structural features, synergy with a combination therapy, and well‐informed indication and patient selection.

BioNTech's Phase 2 trial for melanoma employs candidate BNT111, which differs from Moderna's in multiple ways, including targeting of TAAs instead of neoantigens and use of lipoplex technology. In addition, the selected indication for this trial includes advanced melanomas that are checkpoint‐refractory and/or unresectable. Although TAAs face challenges including susceptibility to central tolerance and risk of autoimmunity, an off‐the‐shelf product suitable for multiple indications would be attractive from manufacturing, cost, and availability perspectives.^70^ BNT111 employs multiple design principles to improve vaccine stability and immunogenicity, including optimization of RNA structure (5′ cap, untranslated regions, and polyA tail), tagging with an MHCI trafficking domain, and inclusion of CD4 helper epitopes. These modifications improve DC activation (via TLR stimulation), mRNA translation, and MHC presentation.^39^, ^71^, ^72^ Analysis of a Phase 1 trial with this candidate showed that 75% of patients developed an immune response to at least one antigen as detected by ELISpot.^72^ Phase 2 results are pending. BioNTech's other named candidates, including BNT112, 113, and 116, target TAAs and viral antigens for indications including HNSCC, NSCLC, and prostate cancer. BioNTech's other named candidate, BNT122, is a personalized neoantigen vaccine for colorectal cancer, however it was not detected in this analysis due to lack of a reference to cancer vaccination in its trial listing.

Overall, most trials are at Phase 1 or 1/2, with 3 at Phase 2 (Figure 4e). The majority of RNA trials use a combination approach (14/19, 73.7%); among all combination therapies identified, 50% were checkpoint blockade antibodies (Figure 4f). ICB therapies are already approved for many of the listed indications for RNA vaccines, including melanoma and HNSCC. Other combinations include standard chemotherapy and radiation.

### 5.3 DNA vaccines

**FIGURE 5:**
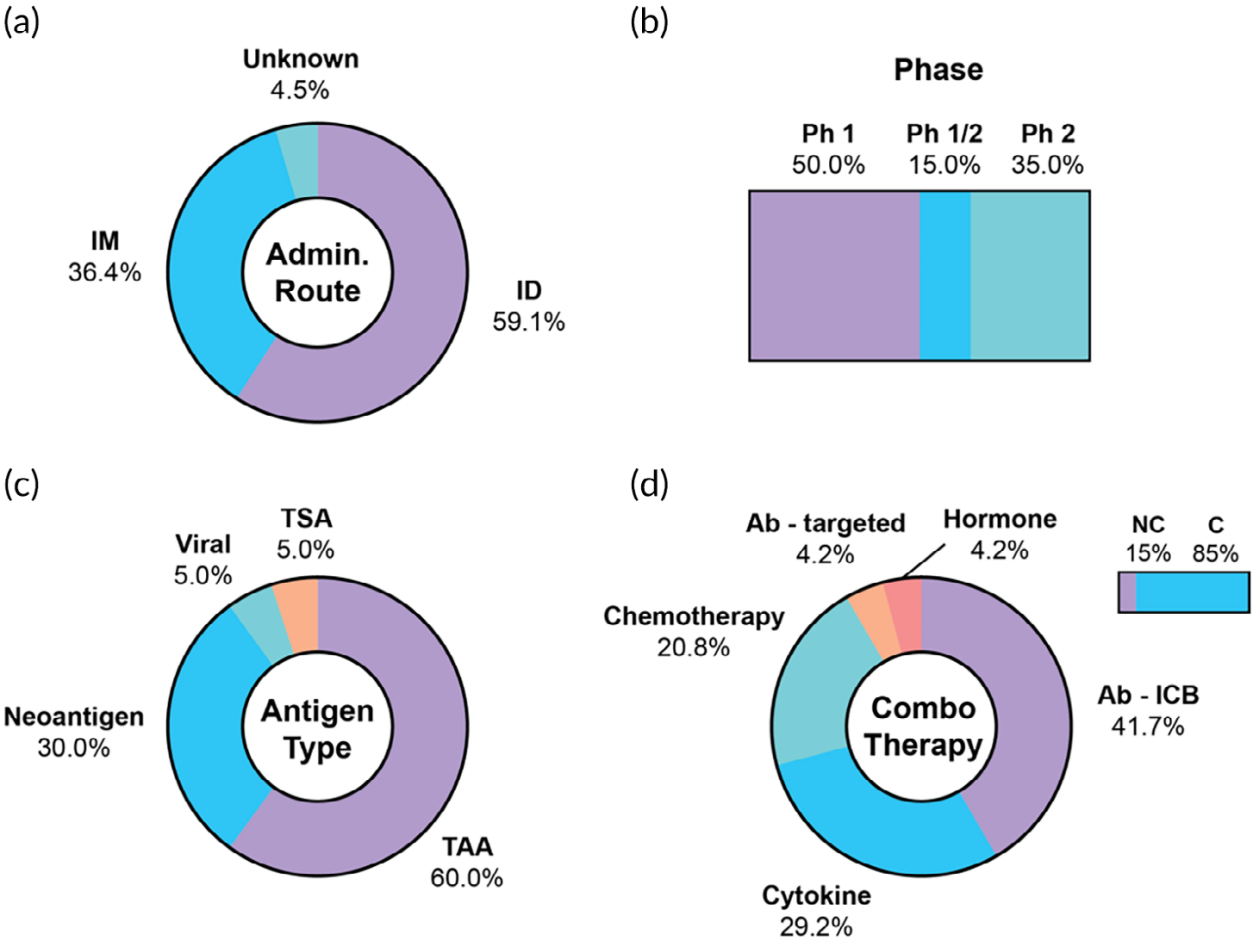
Summary of clinical trials using DNA as antigen material. (a) Routes of administration, (b) trial phases, (c) antigen classification, (d) percentage of trials using a combination therapy (top right bar), and combination therapies by category, among trials using a combination. Ab‐ICB, antibody‐immune checkpoint blockade; Ab‐targeted, antibody‐targeted; C, combination; ID, intradermal; IM, intramuscular; NC, no combination; Ph, phase; TAA, tumor‐associated antigen; TSA, tumor‐specific antigen.

DNA vaccines are designed to induce an immune response by transfecting cells at the injection site with tumor antigens and other costimulatory factors. In this analysis, we identified 20 trials that exclusively employ a DNA approach (Figure 5). Representative trials are listed in Table 3. One additional trial, employing a combination DNA and bacterial vaccine, is discussed in section 5.8. All 20 trials use plasmids as the antigen delivery vehicle. In addition, many trials use more than one plasmid to encode different antigens, along with cytokines and adjuvants. The listed admin routes exclusively include ID (13/22) and IM (8/22), with one unspecified (Figure 5a). Many trials use electroporation of the injection site to enhance intracellular gene delivery and subsequent immunogenicity, as this technique can improve delivery up to 100 to 1000‐fold.^73^ The most common indications for DNA vaccines include breast (18.9%), anogenital (13.5%), prostate (10.8%), and lung cancers (10.8%) (Table S4). While there are no ongoing Phase 3 trials, eight of the 20 trials (40%) are listed at Phase 2 (Figure 5b). For example, WOKVAC is a DNA vaccine that encodes three TAAs and is administered ID with chemotherapy and targeted therapy (NCT04329065, NCT02780401).^74^ Another candidate, INO‐3112 (NCT03439085), is composed of three plasmids expressing the E6 and E7 viral antigens associated with HPV16/18, and the cytokine IL‐12. It is administered IM in combination with the immune checkpoint antibody durvalumab. In a Phase 1 trial for cervical cancer, 6/10 patients generated a T cell response detectable by ELISpot.^75^

**TABLE 3:** Examples of current clinical trials for DNA vaccines.

NCT	Phase	Sponsor/Collaborator	Product name	Indication	Antigen type	Antigens	Route	Combination therapy	Specific combination	Extra details	Other trials
NCT04329065	2	University of Washington	WOKVAC	Breast cancer	TAA	HER2, IGF‐1R, IGFBP2	ID	Antibody‐targeted, chemotherapy	Trastuzumab, pertuzumab, paclitaxel	N/A	NCT02780401
NCT03548467	2	Nykode Therapeutics	VB10.Neo	Skin, lung, kidney, urothelial, head and neck	Neoantigen	N/A	IM	Cytokine	Bempegaldesleukin	CCL3‐tagged neoantigens	NCT05018273
NCT03287427	1	Peter MacCallum Cancer Centre	TetMYB	Colorectal, salivary gland	TAA	MYB	ID	Antibody‐ICB	Tislelizumab	Antigen fused to tetanus toxoid	N/A
NCT03600350	2	University of Wisconsin, Madison	pTVG‐HP	Prostate (non‐metastatic)	TAA	PAP	ID	Antibody‐ICB, cytokine	Nivolumab, GM‐CSF	N/A	NCT02499835, NCT04090528

Abbreviations: ICB, immune checkpoint blockade; ID, intradermal; IM, intramuscular; GM‐CSF, granulocyte‐macrophage colony‐stimulating factor; TAA, tumor‐associated antigen.

Overall, antigens expressed by the DNA vaccines primarily include TAAs (12/20, 60%), the most common being HER2 (breast cancer, three trials), although some utilize a personalized approach with neoantigens (6/20, 30%) (Figure 5c). VB10.Neo, manufactured by Nykode Therapeutics and licensed to Genentech, is a neoantigen DNA vaccine that can hold up to 40 neoantigens, and encodes CCL3 as a targeting element for APC uptake (NCT03548467, NCT05018273).^76^ Overall, the majority of trials (17/20, 85%) use a combination approach (Figure 5d). Of all the listed combination therapies, immune checkpoint blockade is the most common (10/24, 41.7%), followed by cytokines (ex. GM‐CSF, IL‐2) and chemotherapy.

### 5.4 Viral and heterologous vaccines

**FIGURE 6:**
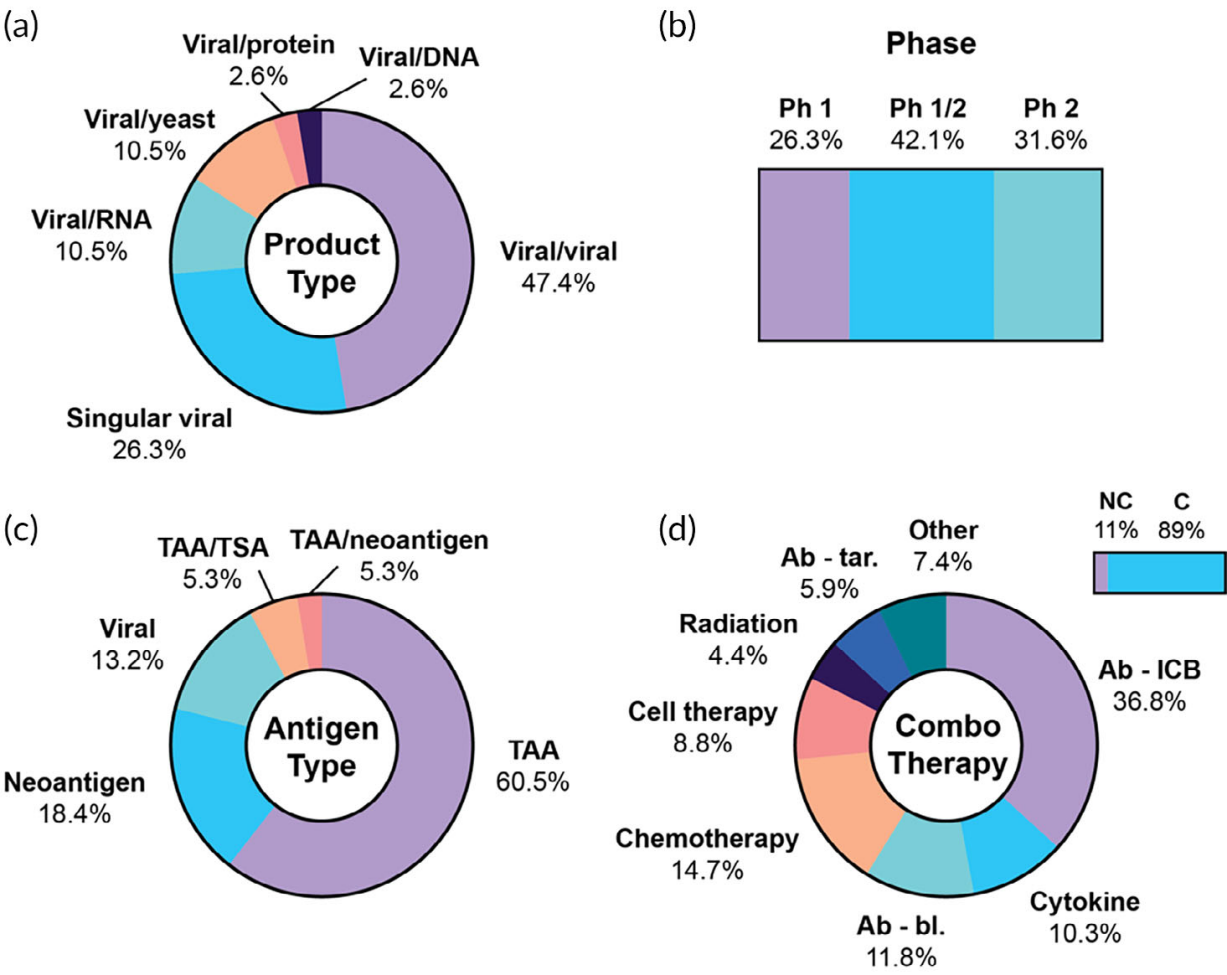
Features of cancer vaccine clinical trials using viral vectors and/or heterologous vaccination schemes. (a) Overview of vaccination strategy, (b) trial phases, (c) antigen classification, (d) percentage of trials using a combination therapy (top right bar), and combination therapies by category, among trials using a combination. Ab‐bl, antibody‐blocking; Ab‐ICB, antibody‐immune checkpoint blockade; Ab‐tar, antibody‐targeted; C, combination; NC, no combination; Ph, phase; TAA, tumor‐associated antigen; TSA, tumor‐specific antigen.

Viral vaccines comprise another major vaccine category, most of which are utilized in heterologous prime‐boost approaches comprising two distinct viral backbones (Figure 6). The benefit of this approach is the avoidance of secondary humoral responses to an immunogenic viral vector, which enhances antigen‐specific T cell proliferation.^42^ A representative selection of trials is listed in Table 4. Of the 38 identified trials, 28 (73.7%) use a combination approach with two different vectors for antigen delivery (Figure 6a). Of these, 18 (64.3%) are dual viral, four viral/RNA, four viral/yeast, one viral/protein, and one viral/DNA. 12 of 38 trials are at Phase 2 (31.6%); one trial was listed at Phase 2/3 (Figure 6b). All four viral/RNA approaches are heterologous prime‐boost regimens registered by Gritstone Bio. Gritstone's lead product combination of GRT‐C901 and GRT‐R902 uses a chimpanzee adenoviral vector encoding patient neoantigens as the priming dose, followed by liposome‐delivered self‐amplifying mRNA encoding those neoantigens as the boosting dose. Gritstone's most advanced trial is a Phase 2/3 trial for metastatic colorectal cancer (NCT05141721), which is buoyed by positive results from a Phase 1/2 trial (GRANITE). Results showed that four of nine patients experienced a molecular response to the therapy, defined by a > 50% reduction in circulating tumor DNA (ctDNA). Median survival in this group exceeded 18 months, compared with 7.8 months among non‐responders, with median overall survival (OS) not yet reached at the time of last report.^77^

**TABLE 4:** Examples of current clinical trials for viral and heterologous vaccines.

NCT	Phase	Sponsor/Collaborator	Product name	Indication	Het.?	Primary construct	Secondary construct	Antigen type	Antigens	Route	Combination therapy	Specific combination	*Other Trials*
NCT05141721	2/3	Gritstone Bio	GRT‐C901, GRT‐R902	Colorectal cancer	Yes	Chimpanzee adenovirus	Self‐amplifying mRNA, via liposome	Neoantigen	N/A	IM, IM	Chemotherapy, antibody‐targeted, antibody‐ICB	Oxaliplatin, fluoropyrimidine, bevacizumab, ipilimumab, atezolizumab	NCT03639714, NCT05456165
NCT05445882	2	National Cancer Institute	BN‐brachyury	Prostate cancer	Yes	Modified vaccinia Ankara (MVA)	Fowlpox	TAA	Brachyury (w/ costim. factors B7‐1, ICAM‐1, LFA‐3)	SC	Cytokine, antibody‐blocking	N‐803, bintrafusp alfa	NCT03493945
NCT03632941	2	Duke University	VRP‐HER2	Breast cancer (recurrent or refractory)	No	Alphavirus‐like replicon particles, derived from VEE virus	N/A	TAA	HER2	IM	Antibody‐ICB	Pembrolizumab	N/A
NCT04990479	1	Nouscom SRL	Nous‐PEV	Melanoma, non‐small cell lung cancer	Yes	Great ape adenovirus (GAd)	Modified vaccinia Ankara (MVA)	Neoantigen	N/A	IM	Antibody‐ICB	Pembrolizumab	N/A

Abbreviations: costim, costimulatory; Het, heterologous, ICB, immune checkpoint blockade; IM, intramuscular; mRNA, messenger RNA; SC, subcutaneous; TAA, tumor‐associated antigen; TSA, tumor‐specific antigen; VEE, Venezuelan equine encephalitis.

Among all viral trials, the majority exclusively target TAAs (23/38, 60.5%), while some target exclusively neoantigens (7/38, 18.4%) (Figure 6c). A few other heterologous prime‐boost approaches target different antigen types with each carrier. A variety of viral scaffolds were identified, the most common being modified vaccinia Ankara (MVA), adenovirus type 5, fowlpox, and chimpanzee adenovirus. For example, the BN‐Brachyury vaccine targeting the prostate cancer TAA brachyury involves two subcutaneous priming doses with a proprietary MVA vector (Bavarian Nordic) and multiple subsequent boosting doses with a fowlpox vector. Both vectors encode the TAA brachyury along with three T cell costimulatory molecules: B7‐1, ICAM‐1, and LFA‐3 (NCT03493945, NCT05445882). Interestingly, it was discovered in preclinical studies that IV administration of these vaccines resulted in improved systemic cytokine production, T cell activation, and NK cell activation, motivating the selection of this administration route.^78^ In a Phase 1 trial, 9/13 patients exhibited a T cell response to brachyury, and 7/11 analyzed developed CD8 T cell responses to CEA and MUC1, indicating antigen spreading.^79^

Overall, indications for the viral‐based cancer vaccines are diverse, the most common being prostate (12.9%), gastric/gastroesophageal junction (GEJ)/bowel (11.4%), and colorectal (11.4%). Of the 38 trials, 34 (89.5%) include a therapeutic combination (Table S5). Among those trials, the most common intervention is ICB (25/68, 36.8%), followed by chemotherapy, blocking antibodies, and cytokines (Figure 6d). Blocking antibodies include bevacizumab, which targets vascular endothelial growth factor (VEGF), and bintrafusp alfa, an investigational bispecific protein targeting TGF‐β and PD‐L1.^80^

### 5.5 DC/APC vaccines

**FIGURE 7:**
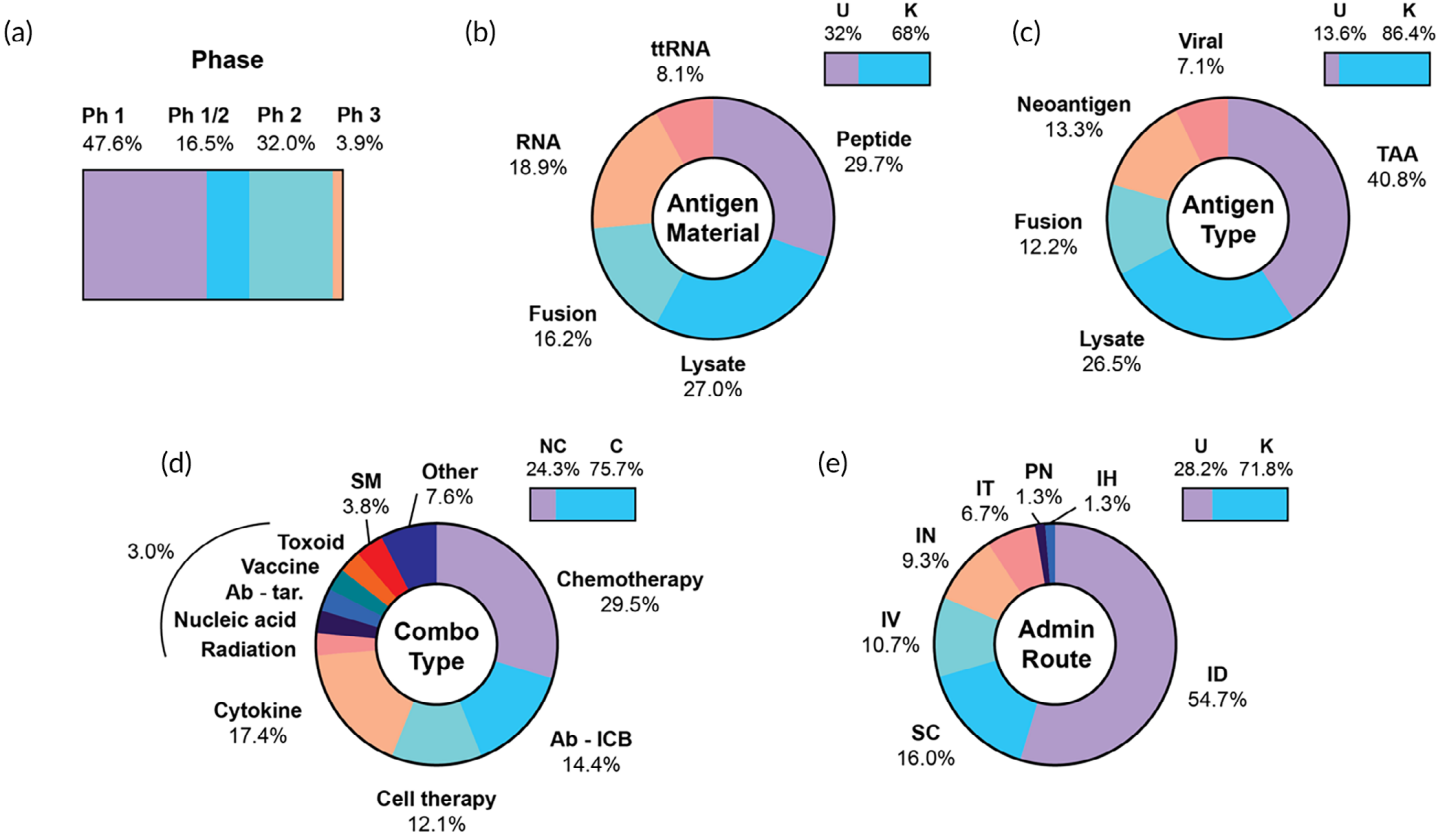
Overview of clinical trials using antigen‐presenting cell vaccines. (a) Trial phases, (b) percentage of trials with a known material used to deliver antigen to cells ex vivo (top right bar), and summary of materials, (c) percentage of trials with a known antigen classification, and summary of antigen types (d) percentage of trials using a combination therapy (top right bar), and combination therapies by category, among trials using a combination, (e) percentage of trials with a known route of administration (top right bar), and breakdown of routes. Ab‐ICB, antibody‐immune checkpoint blockade; Ab‐tar, antibody‐targeted; C, combination; K, known; ID, intradermal; IH, intrahepatic; IN, intranodal; IT, intratumoral; IV, intravenous; NC, no combination; Ph, phase; PN, perinodal; SC, subcutaneous; SM, small molecules; TAA, tumor‐associated antigen; ttRNA, total tumor RNA; U, unknown.

Antigen‐presenting cell (APC) vaccines comprise the second‐largest subclass of cancer vaccines, second only to peptide vaccines, with 108 references in clinical trial listings (Figure 1). Of these, 103 are listed as DC, one as APC, one as PBMC, and three as monocytes. Although all of these products contain cells with antigen‐presenting functions, DCs are the classical antigen‐presenting cells and the subject of most clinical investigations (Figure 7). Hereafter, the terms DC and APC may be used interchangeably. A representative selection of trials can be found in Table 5. The most common indication category for DC vaccines is the brain (30/127, 23.6%); these trials almost exclusively were for treatment of glioblastoma (GBM) (Table S6). 64.1% of trials are in Phase 1 or 1/2, with almost one third (32.0%) at Phase 2, and four trials (3.9%) at Phase 3 (Figure 7a). Of the Phase 3 trials, three are indicated for GBM and one for uveal melanoma. Antigen priming materials in these trials include tumor lysate, autologous total tumor RNA, and a tumor cell fusion (NCT03400917, NCT04277221, NCT00045968, NCT01983748).

**TABLE 5:** Examples of current clinical trials for DC/APC vaccines.

NCT	Phase	Sponsor/Collaborator	Product Name	Indication	Antigen material	Antigen type	Antigens	Route	Combination therapy	Specific combination	Extra details	Other trials
NCT05100641	3	Aivita Biomedical	AV‐GBM‐1	Glioblastoma	Lysate (autologous)	Lysate	N/A	SC	Chemotherapy, cytokine	Temozolomide, GM‐CSF	N/A	NCT03400917
NCT01983748	3	University Hospital Erlangen	DCaT‐RNA	Uveal melanoma	TTRNA (autologous)	Lysate	N/A	IV	None	N/A	N/A	N/A
NCT03059485	2	Dana‐Farber Cancer Institute	DC/AML	AML (in remission)	Tumor fusion (autologous)	Tumor fusion	N/A	Unspecified	None	N/A	N/A	NCT03679650, NCT01096602
NCT03387553	1	H. Lee Moffitt Cancer Center and Research Institute	DC1	Breast cancer	Unknown	TAA	HER2	IN	Chemotherapy, surgery	TCHP	N/A	NCT02063724, NCT05378464, NCT02061423, NCT03384914, NCT05325632
NCT04912765	2	National Cancer Centre, Singapore	N/A	Liver cancer, colorectal cancer	Peptide	Neoantigen	N/A	ID	Antibody‐ICB	Nivolumab	N/A	N/A
NCT05127824	2	University of Pittsburgh	alpha‐DC1/TBVA	Clear cell renal carcinoma	Peptide	TAA	Tumor blood vessel antigens	ID	Chemotherapy, surgery	Cabozantinib	Alpha type 1‐polarized DC	N/A
NCT01946373	1	Karolinska University Hospital	N/A	Melanoma (metastatic)	Lysate, peptide	Lysate, TAA	NY‐ESO‐1	ID	Cell therapy, cytokine	T cells (TIL), IL‐2	N/A	N/A
NCT03371485	1	Cancer Research UK	AST‐VAC2	NSCLC	Unknown	TAA	hTERT	ID	None	N/A	Allogeneic, ESC‐derived DC	N/A

Abbreviations: AML, acute myeloid leukemia; APC, antigen‐presenting cell; DC, dendritic cell; ICB, immune checkpoint blockade; ID, intradermal; IN, intranodal; IV, intravenous; GM‐CSF, granulocyte‐macrophage colony‐stimulating factor; NSCLC, non‐small cell lung cancer; SC, subcutaneous; TAA, tumor‐associated antigen; TIL, tumor‐infiltrating lymphocyte; TTRNA, total tumor RNA.

Aside from the brain, the most common indications for DC vaccines include cancers of the blood, lung, skin (melanoma), and breast. While few vaccines in the overall space are indicated for liquid cancers, this was the second most common indication for DC vaccines (15/127, 11.8%). One of the more advanced candidates for liquid cancers is a tumor fusion vaccine indicated for patients in remission from acute myeloid leukemia (AML), and it is designed to prevent cancer recurrence. This candidate, known as DC/AML, is being investigated in multiple Phase 2 trials (NCT03059485, NCT01096602, NCT03679650).

An important consideration in DC vaccine development is the material used to load cells with the target antigen(s) ex vivo. Among trials with a listed material, peptides and lysate are the most frequently used, followed by RNA, tumor fusions, and total tumor RNA (Figure 7b). The lysate, tumor fusion, and total tumor RNA approaches (combined 43% of all approaches) rely on the diversity of antigens present in biopsied tumor cells to initiate an immune response, as opposed to priming with defined antigens or neoantigens. The majority of approaches for defined antigens target TAAs (40.8% of known antigen listings), the most common including WT1 (leukemia) and HER2 (breast cancer), along with survivin, hTERT, and NY‐ESO‐1 (various solid tumors) (Figure 7c, Table S7). A small fraction of trials includes viral targets, particularly for HPV‐ and cytomegalovirus (CMV)‐associated cancers. Personalized neoantigen targets are also making their way into the DC space; 13.3% of trials use DCs loaded with neoantigen peptides. Although the standard cell source for DC generation is monocytes derived from autologous PBMCs and nearly all trials use autologous cells (99/103 trials, 96.1%), we identified four trials using allogeneic cells (NCT03371485, NCT03970746, NCT04739527, NCT03697707).

The majority of DC trials utilize a therapeutic combination (78/103, 75.7%) (Figure 7d). Among these trials, chemotherapy is the most common (39/132, 29.5%), owing to the large proportion of trials targeting GBM, for which maintenance temozolomide chemotherapy is usually employed in combination with novel interventions. Cytokines are another common combination (23/132, 17.4%), due to the frequent addition of GM‐CSF to promote the survival and function of injected DCs. Interestingly, cell therapies (16/132, 12.1%) are used as combinations about as frequently as ICB (19/132, 14.4%), in contrast to peptide and other categories which employ ICB much more frequently. Listed cell therapies include various types of T cells, including chimeric antigen receptor (CAR) T cells, tumor‐infiltrating lymphocytes (TILs), and cytokine‐induced killer cells (CIK). These striking differences are likely due to the prevalence of GBM and liquid indications, for which ICB is not usually effective.^81^, ^82^ The prevalence of T cell combinations is likely driven by the potential for the administered DC product to directly interact with and further expand T cells in the lymph nodes. Administration routes are highly variable, the most common being ID (Figure 7e).

### 5.6 Tumor cell vaccines

**FIGURE 8:**
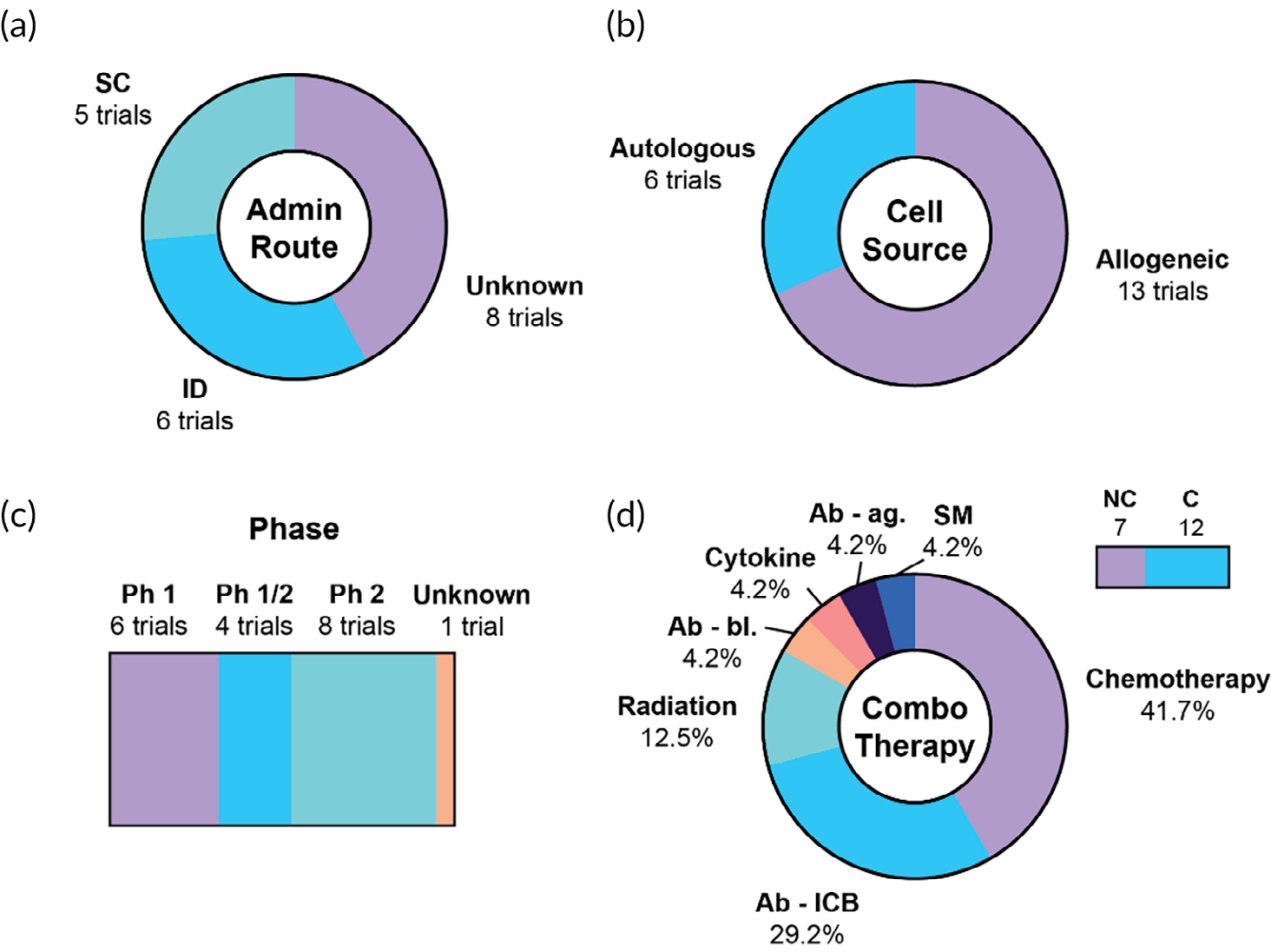
Summary of clinical trials using tumor cell vaccines. (a) Routes of administration, (b) nature of cell source, (c) overview of trial phases, (d) number of trials using a combination therapy (top right bar), and combination therapies by category, among trials using a combination. Ab‐ag, antibody‐agonist; Ab‐bl, antibody‐blocking; Ab‐ICB, antibody‐immune checkpoint blockade; ID, intradermal; Ph, phase; SC, subcutaneous; SM, small molecule.

Tumor cell‐based vaccines are among the earliest cancer vaccines evaluated in human. However, they currently comprise only 5.3% (19/360) of all identified trials (Figure 8). Tumor cell‐based approaches are the most homogenous of all modalities, as all identified trials employed variations of GVAX, a cellular vaccine that has been under intense clinical evaluation for decades. This vaccine consists of autologous or allogeneic tumor cells typically transduced with adenovirus to express GM‐CSF, an APC‐attracting and ‐activating cytokine, and lethally irradiated to prevent tumor cell proliferation within the patient. All 19 trials use genetically modified tumor cells, and 15 of these (78.9%) express GM‐CSF (Table S8). Of reported routes, all are injected SC or ID (Figure 8a). 6/19 trials (31.6%) use autologous cells from patient biopsy material, and the remaining 13/19 (68.4%) use allogeneic cells in an off‐the‐shelf approach (Figure 8b). Nearly half (8/19) of the identified trials are indicated for pancreatic cancer (Table S9). In addition, 8/19 trials are listed at Phase 2, although none are listed at Phase 3 (Figure 8c). Of all the trials, 12/19 (63.2%) use a combination approach, the most common interventions being chemotherapy (41.7%) and ICB (29.2%) (Figure 8d). Chemotherapies are often standard‐of‐care for these patients and thus essential for ethical clinical trial design, and ICB has the potential to ameliorate exhaustion of vaccine‐generated antigen‐specific T cells.

### 5.7 Other vaccines

**FIGURE 9:**
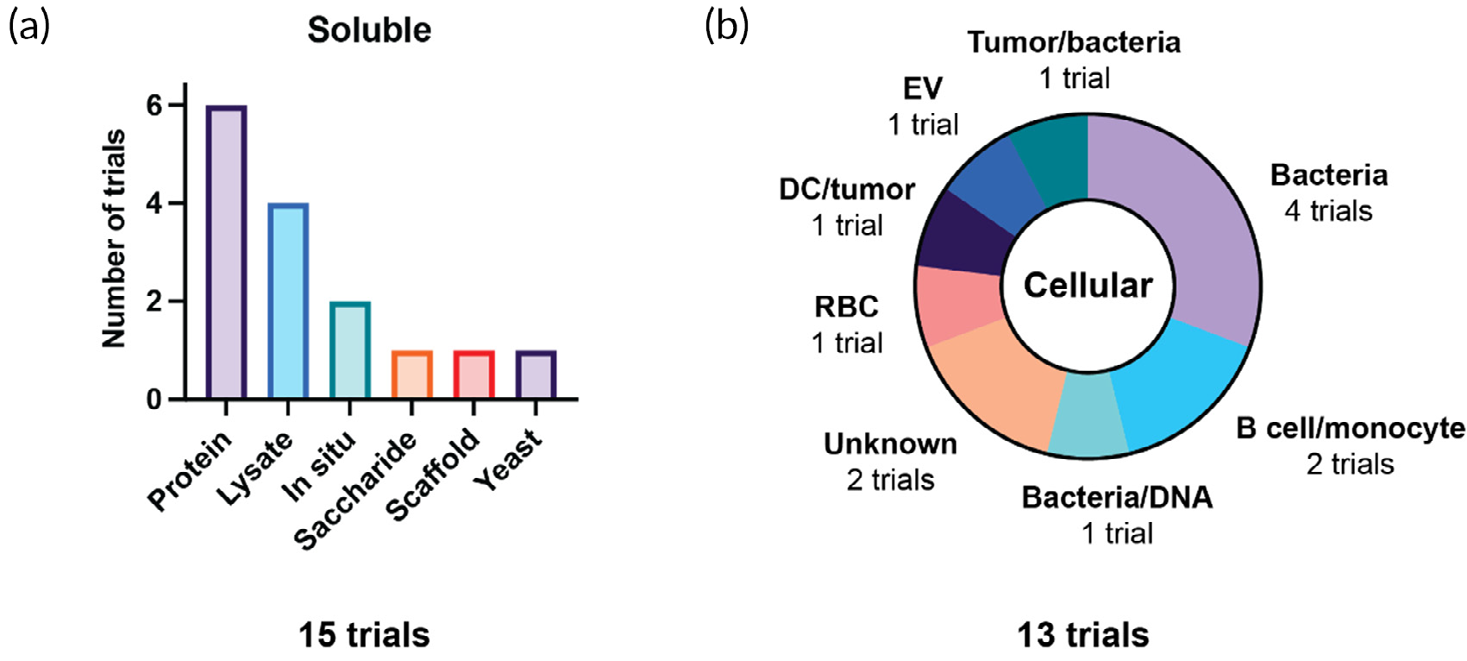
Overview of other vaccine types. (a) Antigen materials used in soluble vaccines, (b) types of cells used in novel cellular vaccines. DC, dendritic cell; EV, extracellular vesicle; RBC, red blood cell.

We identified a total of 28 trials that did not fit into the given subcategories, including 13 cellular vaccine trials and 15 soluble antigen‐based trials (Figure 9). Among soluble vaccines, a few trials employ whole proteins (6/15) or tumor lysate (4/15) as the primary vaccine material (Figure 9a). Protein vaccines include HPV antigens and some novel fusion proteins to enable cell targeting or immunogenicity. OBI‐822 (OBI Pharma) consists of a hexasaccharide antigen (Globo H) linked to the adjuvant keyhole limpet hemocyanin (KLH); it is the only saccharide antigen identified in this analysis. OBI‐821 is being investigated in a Phase 3 trial for triple‐negative breast cancer (TNBC) in combination with standard‐of‐care chemotherapy (NCT03562637). The other carriers include a poly(lactic‐co‐glycolic acid) (PLGA) scaffold loaded with melanoma lysate and GM‐CSF, and a yeast vaccine carrying tumor lysate. Among the cellular vaccine trials, 5/15 use bacteria (Figure 9b). These approaches involve the transformation of bacterial cells with tumor antigens, enabling antigen presentation in an inherently immunogenic context. For example, ADXS11‐001 (Advaxis, Inc.) consists of an attenuated *Listeria* strain expressing the HPV‐associated protein E6, for vaccination against HPV‐associated malignancies (NCT02002182). Other cellular vaccine trials employ combinations of different cell types together, including DCs, tumor cells, and bacterial cells.

### 5.8 Prophylactic vaccines

**FIGURE 10:**
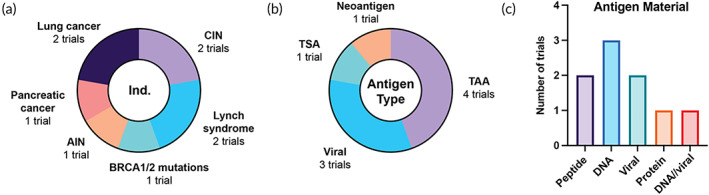
Features of vaccine trials used for prophylactic applications. (a) Indications/conditions, (b) antigen classifications, (c) antigen materials. CIN: cervical intraepithelial neoplasia, AIN, anal intraepithelial neoplasia; TAA, tumor‐associated antigen; TSA, tumor‐specific antigen.

Cancer prophylaxis via vaccination is an attractive approach, especially for patients who have premalignant lesions or are at high risk for developing certain cancers due to genetic or environmental factors. In this analysis, we excluded approved nonavalent vaccines used for the prevention of HPV‐related cancers and/or treatment of HPV‐related neoplasia (pre‐cancer), due to their widespread clinical use worldwide. In total, we identified nine trials with a prophylactic use case (Figure 10).

Six trials are indicated to prevent cancer in high‐risk patients, and three to prevent the progression of existing neoplastic lesions. We identified two trials for HPV‐associated cervical intraepithelial neoplasia that use novel approaches, including a DC vaccine and a DNA vaccine delivered IM via electroporation (Figure 10a). Both vaccines target the HPV‐associated proteins E6 and E7 and are currently in Phase 1 trials. Lynch syndrome is a hereditary disease that affects about one in 300 individuals and results in a high risk of colorectal cancer. We identified two prophylactic vaccines for Lynch syndrome patients, including a DNA vaccine targeting common TAAs (NCT05419001) and a heterologous viral vaccine targeting patient‐specific neoantigens (NCT05078866). Two trials are indicated for the prevention of lung cancer in smokers, including a MUC1‐targeted peptide vaccine with poly‐ICLC adjuvant (NCT03300817) and a whole protein vaccine designed to induce a humoral immune response to the TAA EGF (NCT04298606). Other indications in this category include pancreatic cancer, breast cancer, and anogenital cancers. The trials for anogenital and cervical cancers have viral targets (HPV), while most of the remaining trials target TAAs (4/9, 44.4%) (Figure 10b). The antigen materials used are highly variable and encompass all identified soluble categories; no cellular vaccines were identified (Figure 10c).

## 6 DISCUSSION, OPPORTUNITIES, AND CHALLENGES

Despite the increasing clinical interest in cancer immunotherapy, many of the recent cancer vaccine clinical trials have reported disappointing results, especially at Phase 2 and 3 stages. While the efficacy of cancer vaccines as assessed by cancer progression or survival does not currently rival that of conventional therapies, significant progress has been made towards eliciting tumor antigen‐specific immune responses. Furthermore, the lengthy clinical development process for cancer vaccines and rapid preclinical progress may paint the clinical field as outdated. However, recent advancements in data analysis and experimental tools have elucidated many critical mechanisms involved during the immune responses induced by immunotherapies, providing key insights to guide future therapeutic vaccination approaches. We highlight several key points regarding ongoing challenges and future directions in the field of cancer vaccines.

### 6.1 Delivery carriers and novel formulations

Antigens have a short half‐life when in soluble form, limiting their ability to initiate immune responses. To protect from enzymatic degradation and provide prolonged availability to the immune cells, antigens are often encapsulated in a diverse range of delivery carriers. Nanoparticles have been thoroughly explored as vaccine delivery vehicles. The possibility of co‐loading antigens and adjuvants to bolster vaccine efficacy, enhancing cytosolic delivery of antigen cargo to APCs, and promoting efficient antigen drainage to the lymphoid organs to target antigen‐presenting cells render the use of nanoparticles an appealing strategy for vaccine delivery. The COVID‐19 outbreak fueled the advancement of long‐studied lipid‐based nanoparticle delivery carriers to the clinic and eventual approval. In fact, many of those vaccine platforms were originally developed for other indications including cancer vaccines, and many other materials, such as metals, silica, polymers, etc., are under preclinical investigation for use as cancer vaccines.^83^, ^84^ Nanoparticles can also be rationally designed to harness functional immune‐stimulatory properties of the constituent materials at the nanoscale. Such nanoparticles have been extensively discussed in many reviews.^85^, ^86^, ^87^

Despite these possibilities, clinical translation of nanoparticles remains a major challenge. While lipid nanoparticles containing mRNA have seen some promising successes, other formulations remain heavily underexplored in the clinic. The reasons for this phenomenon can be segmented into two general buckets. From a technical perspective, it has historically been difficult to develop formulations with adequate stability, biocompatibility, and antigen and adjuvant co‐delivery and release. From a clinical perspective, the issues of manufacturing at scale and lack of modularity have hindered real‐world study and application of nanoparticle cancer vaccines.^88^, ^89^

One major obstacle is the synthesis and scale‐up of complex formulations containing multiple components, which impedes treatment of large cohorts in clinical trials and in the general patient population. Similarly, formulation constraints surrounding physicochemical properties of the constituent materials severely limit formulation modularity, further constricting the pool of eligible patients. One approach to help bypass the complexity of nanoparticles is the development of molecular conjugates, which can co‐localize signal presentation and/or modulate lymph node trafficking kinetics. For example, tagging of various vaccine cargoes with amphiphilic lipid tails improved lymph node delivery via albumin hitchhiking in vivo, enhancing the generation of antigen‐specific CD8+ T cells.^90^ Similarly, conjugation of diverse antigen peptides directly to albumin has a similar effect.^91^ Another strategy is to facilitate spontaneous nanoparticle assembly via chemical modification of components or inclusion of supramolecular materials. In an approach leveraging both conjugation and self‐assembly, peptides conjugated to TLR7/8 agonists and self‐assembled into 20 nm particles were shown to boost T cell immunity and tumor eradication.^92^ Importantly, the platform was compatible with diverse neoantigen peptides of differing properties.

Biomaterial scaffolds are one promising option to address many of the limitations with nanoparticles.^93^, ^94^ Once loaded with immunomodulatory reagents and injected or implanted, these serve as an immune‐stimulating depot, providing signals to innate immune cells to initiate the immune response against cancer. Biocompatibility, persistence of the depot and released therapeutics, manufacturing, and potential to induce chronic inflammation are factors that need to be thoroughly investigated before clinical application. Recent preclinical work has showed that components of diverse properties, including proteins and peptides, small molecules, and cytokines, may be co‐loaded in macroporous cryogels and effectively released to stimulate prophylactic and therapeutic responses against cancer.^95^ In particular, the inclusion of GM‐CSF promotes infiltration of APCs to the site containing antigen and adjuvant, leading to enhanced immune responses. Another material platform composed of injectable, self‐assembling mesoporous silica rods coated with polyethyleneimine (PEI) was shown to load diverse peptide cargos via simple, rapid adsorption to PEI.^96^ This approach lends modularity to an otherwise complex system, which may better facilitate clinical translation.

### 6.2 Antigen discovery and optimization

Vaccines targeting multiple epitopes have many advantages over those that target a single epitope. Tumor cells are likely to lose expression or presentation of a single antigen under significant selective pressure, especially if the antigen in question is not the result of a driver mutation. Immune escape by tumors is thought less likely to occur when a vaccine targets multiple epitopes.^97^ Initial multiple‐epitope vaccines were traditionally prepared from tumor cell lysates obtained from patient biopsies, but resulting immune responses were often suboptimal due to low quantities of immunogenic antigens. In contrast, delivery of recombinant or synthetic antigens improves the quality of immune responses by delivering the antigen with determinate specificity and in a sufficient quantity.^98^ With recent advances in computation and machine learning processes, great progress has been made towards identification of patient‐specific, immunogenic neoantigens.^62^, ^99^ These advances, together with high‐throughput manufacturing technologies, make it possible to include many defined neoantigens in a vaccine, which increases the likelihood of generating more robust immune responses. In addition, providing multiple epitopes may elicit different types of immune responses, such as CD8 T cell, CD4 T cell, and B cell responses, which may cooperate to improve therapeutic outcomes.^28^ Overall, through carefully chosen antigen types, cellular targets, and combinations, there is great potential to improve vaccine efficacy and advance clinical applicability.

### 6.3 Adjuvants

The majority of the cancer vaccine types investigated here either exploit inherently immunogenic carriers (e.g., nucleic acids, viruses) and therefore do not require adjuvants. However, most peptides are weakly immunogenic. As a result, they often require support by adjuvants to achieve robust immunity encompassing potent humoral and cellular responses and long‐lived memory.^90^, ^100^ Among peptide vaccine trials identified here, only two‐thirds (66%) listed an adjuvant, which may result in part from incomplete reporting. Among the trials that listed an adjuvant, the synthetic RNA analog poly‐ICLC and emulsion‐forming adjuvant Montanide were most common. Poly‐ICLC is a double‐stranded RNA analog that activates APCs via TLR3, and has been studied for decades for its ability to enhance T cell responses.^101^ The mechanism of action for emulsion‐based adjuvants like Montanide is less clear. While these agents were once thought to enhance immune responses by prolonging vaccine retention at sites of injection, more recent evidence suggests that they drive a local inflammatory response, resulting in vaccine uptake by APCs and transport to the lymph nodes.^102^, ^103^, ^104^ Other adjuvants identified in this analysis included the small molecule TLR8 agonists resiquimod and imiquimod, keyhole limpet hemocyanin (conjugated to the antigen), tetanus toxoid, unspecified oligonucleotides, and the saponin adjuvant OPT‐821. The latter class of adjuvants, saponins, can be assembled into nanoparticles in the presence of cholesterol and phospholipids. Multiple saponin‐containing adjuvants are now FDA‐approved and utilized in vaccines against infectious diseases; although their mechanism of action remains unclear, monocytes and macrophages are believed to play an important role in the immune response.^105^, ^106^ However, like all other adjuvants, they have yet to achieve FDA approval in oncology. Overall, the field is still lacking adequate information to effectively select vaccine adjuvants and develop effective formulations.

Another function of the selected adjuvant is to tune the type and quality of the resulting immune response. Alum, a widely‐used adjuvant in infectious disease vaccines is known to trigger a Th2‐skewed immune response, whereas a Th1 immune response either alone or paired with a Th2 response is generally favored for the treatment of cancer, demonstrating the need for careful choice of adjuvants during vaccine formulation for clinical use.^107^ Another strategy to bolster the effectiveness of adjuvants is through the combination of multiple adjuvants which activate different immune pathways, potentially leading to more robust immune responses.^26^, ^108^ Combined adjuvants have already been extensively studied in the vaccinology field, the idea and advantages of which can readily be adopted for cancer vaccines.^104^ While TLR‐activating adjuvants are common in the clinical space, those that target other pathways, such as cyclic GMP‐AMP synthase stimulator of interferon genes (cGAS‐STING), Nod‐like receptor (NLR), and Rig‐I‐like receptor (RLR), are emerging as promising options for adjuvant combinations. The cGAS‐STING pathway is a cytosolic DNA sensing pathway of particular interest, since in recent years it has been shown to play a major role in tumor control.^109^, ^110^, ^111^ Agents that act on this pathway stimulate type I interferon production in tumors, which drives downstream events including maturation of critical subsets of APCs (cDC1s), recruitment of T cells, and cross‐presentation to generate effective CD8+ T cells. However, their instability, poor membrane permeability, and toxicity have precluded their effective use as systemic agents. Novel delivery carriers and complexes may be able to overcome this challenge in the future.^112^, ^113^, ^114^, ^115^

### 6.4 Advances in cell‐based vaccination

APC vaccines offer an alternative way to control immune‐activating signals. While the ability to prime cells ex vivo with antigens and maturation stimuli is an attractive feature, these vaccines have multiple limitations. One major challenge is effective and timely cross‐presentation of antigen peptides. moDCs do not cross‐present antigens efficiently, and while they may be incubated ex vivo with peptides before infusion, peptide–MHC complexes are rapidly recycled, leading to suboptimal immune responses. An ideal vaccine would consist of bona fide cDC1s, an APC subset capable of cross‐presentation.^12^, ^13^, ^14^, ^21^ However, these cells are too rare to isolate from the bloodstream, and also difficult to recapitulate ex vivo using monocytes as starting material. Even so, in vitro‐cultured cDC1 showed superior efficacies compared with conventionally used monocyte‐derived DCs.^116^, ^117^ Administration route is also an important consideration that has yet to be fully optimized in the clinic. For example, DCs administered SC or IV result in differential cell trafficking and organ accumulation profiles, leading to varied efficacies.^118^, ^119^, ^120^ The use of other innate immune cell types warrants investigation, especially for the treatment of cancers for which DC vaccines are less effective. For example, there have been reports of unique tumor‐homing behavior of IV‐administered macrophages, allowing them to be used as a cancer diagnostic.^121^ There also has been a case of macrophages being used as a gene delivery carrier to the brain, which demonstrates the potential of macrophages to be used as a vaccine against brain cancer.^122^ Exploration of various cell types and phenotypes may provide potential solutions to cancers that are not suited for treatment with currently available vaccines.

While tumor cell‐based vaccines have been investigated for over 30 years, they have yet to demonstrate sufficient efficacy in a pivotal trial. Although they contain a diverse and often patient‐specific array of antigens, their immunogenicity is often insufficient. Multiple new strategies are under development at the preclinical stage to reinvigorate this field. One promising strategy to enhance immunogenicity is functionalization of tumor cells or tumor cell membrane components with pathogens or components thereof. For example, tumor cells preserved in silica and functionalized with TLR ligands improved DC uptake and activation and delayed tumor growth in murine models of ovarian cancer.^123^ In another approach, extracted membranes from tumor cells and *E. coli* were fused to create a nanoparticulate vaccine containing tumor cell‐derived antigens and pathogen‐associated molecular patterns (PAMPs) found on bacterial membranes.^124^ Finally, exosomes derived from tumor cells are an additional option, as they possess excellent biocompatibility and stability along with a capacity to carry therapeutic drugs of varying properties.^125^, ^126^

### 6.5 Other limitations and combination treatments

In general, the limitations experienced by cancer vaccines reflect those plaguing the immunotherapy field as a whole. For therapies that rely on an antigen‐specific immune response, the identification of immunogenic antigens is an ongoing challenge. In addition, tumor‐intrinsic processes including downregulation of MHCI expression and immune editing may result in antigen loss, which limits the efficacy of cancer vaccines.^127^, ^128^ One novel approach to bypass this challenge is vaccination against pan‐cancer targets that are not restricted by MHC presentation. Such targets are believed to be byproducts of cellular stresses and changes in metabolism that occur in tumor cells.^129^ In a recent preclinical study, Badrinath et al. developed a scaffold vaccine targeting MICA and MICB, DNA damage‐associated proteins which are expressed by many cancers and recognized by NK cells and some T cells.^130^ In the tumor microenvironment, proteolytic cleavage of MICA/B by cancer‐associated proteases limits their recognition by effector cells and subsequent tumor cell killing. The mesoporous silica rod scaffold vaccine containing polyvalent protein antigen, GM‐CSF, and the adjuvant CpG ODN 1826 resulted in robust generation of MICA/B antibodies, blocking proteolysis of surface‐associated MICA/B and enabling elimination of tumor cells. Further efforts are underway to systematically identify other conserved targets that may one day be used to produce cancer vaccines that are not limited by immune evasion or patient HLA type.

Another limiting factor for immunotherapies is tumor heterogeneity. Properties such as driver mutations, antigen expression, stromal characteristics, and metabolic features can vary widely both within a single tumor and more broadly within a patient population, and all may have substantial effects on responses to therapy.^131^ The adoption of patient‐specific neoantigens as cancer vaccine targets is a step in the right direction, in this regard. Finally, one of the most important obstacles in the immunotherapy field is systemic and tumor‐specific immunosuppression.^132^, ^133^ While vaccines may be capable of generating CD8+ effector T cells against vaccine antigens, their efficacy is often hindered due to poor tumor infiltration and limited cytotoxicity in the immunosuppressive tumor microenvironment. This property is a major factor governing the application of therapeutic combinations. When administered in the right context, combination therapies may promote cooperation with humoral immunity, development of more diverse responses via antigen spreading, and effector cell infiltration and killing.

The major categories of combination treatments identified in the analysis include ICB, chemotherapy, cytokines, and antibodies. Treatments that target tumor cells directly such as chemotherapy drugs and radiation, are known to trigger cell death within the TME, causing the secretion of danger signals that lead to immune cell infiltration. This process induces the transition of the tumor into a so‐called ‘hot’ state, favoring further waves of immune cell infiltration that contribute to stronger responses to immunotherapies.^134^, ^135^ On the other hand, there are immunomodulatory drugs, mainly antibodies, that target immune cells through receptors such as CD40, 4‐1BB, OX40, and PD‐1.^136^, ^137^, ^138^, ^139^, ^140^ These treatments work by blocking inhibitory signals or sustaining the activity and/or proliferation of immune cells to dampen the effects of immune suppression exerted by the TME and ultimately prompt more potent or durable humoral and cellular immune responses.^141^, ^142^ Finally, cytokine therapies function by recruiting cells to a certain site, promoting survival, or inducing pro‐inflammatory, anti‐tumor functions. Although cytokines are potent therapies, their practical application remains a challenge due to concerns with toxicity and dosing regimen optimization. To achieve a better selection of treatment regimen for individual patients, clinical failures and successes should be carefully acknowledged, followed by the categorization of patient groups based upon previous lines of treatment, cancer type, and tumor profile.

## 7 CONCLUSION

The analysis presented here underscores a diverse, evolving landscape of vaccines in oncology. The primary vaccine types under investigation include peptides, RNA, DNA, tumor cells, viral vectors, and cellular (DC/APC) vaccines. Clinical responses generated by cancer vaccines are heavily governed by indication, antigen selection, patient‐specific factors, vaccine type, and combination therapies. Our evaluation of ongoing clinical trials indicates that adept attention to all these factors will be a major determinant of the future success of cancer vaccines. Because many cancer vaccine trials often take years to proceed from each clinical phase to the next, the current analysis does not yet reflect many promising preclinical interventions, including novel adjuvants and drug delivery carriers. Although clinical results have been disappointing to date, the latest data indicate that there may be a more permanent niche for vaccines in the expanding repertoire of therapeutic interventions against cancer.

## AUTHOR CONTRIBUTIONS

**Morgan Janes:** Data curation (equal); formal analysis (equal); investigation (equal); methodology (equal); visualization (equal); writing – original draft (equal); writing – review and editing (equal). **Alexander Gottlieb:** Data curation (equal); methodology (equal); writing – original draft (equal); writing – review and editing (equal). **Kyung Soo Park:** Writing – original draft (equal); writing – review and editing (equal). **Zongmin Zhao:** Conceptualization (equal); writing – review and editing (equal). **Samir Mitragotri:** Conceptualization (equal); funding acquisition (lead); writing – review and editing (equal).

**Abbreviations**
APC: antigen‐presenting cell
AML: acute myeloid leukemia
BCG: Bacillus Calmette‐Guérin
CAR: chimeric antigen receptor
CCL3: chemokine ligand 3
cDC1: conventional type I dendritic cell
CIK: cytokine‐induced killer
cGAS: cyclic GMP‐AMP synthase
CMV: cytomegalovirus
COVID‐19: coronavirus disease 2019
CRC: colorectal cancer
ctDNA: circulating tumor DNA
DC: dendritic cell
*E. coli*
: *Escherichia coli*
EGF: epidermal growth factor
ELISpot: enzyme‐linked immunosorbent spot
EMA: European Medicines Agency
FDA: Food and Drug Administration
GBM: glioblastoma
GEJ: gastroesophageal junction
GM‐CSF: granulocyte‐macrophage colony‐stimulating factor
HNSCC: head and neck squamous cell carcinoma
HPV: human papillomavirus
ICB: immune checkpoint blockade
ID: intradermal
IL‐12: interleukin‐12
IL‐2: interleukin‐2
IM: intramuscular
IV: intravenous
KLH: keyhole limpet hemocyanin
LNP: lipid nanoparticle
MHC: major histocompatibility complex
moDC: monocyte‐derived dendritic cell
mRNA: messenger RNA
MVA: modified vaccinia Ankara
NK: natural killer
NLR: Nod‐like receptor
NSCLC: non‐small cell lung cancer
OS: overall survival
PBMC: peripheral blood mononuclear cell
PEI: polyethyleneimine
PLGA: poly(lactic‐co‐glycolic acid)
PRR: pattern recognition receptor
RCC: renal cell carcinoma
RLR: Rig‐I‐like receptor
SC: subcutaneous
SLP: synthetic long peptide
STING: stimulator of interferon genes
TAA: tumor‐associated antigen
TIL: tumor‐infiltrating lymphocyte
TLR: Toll‐like receptor
TME: tumor microenvironment
TNBC: triple‐negative breast cancer
TSA: tumor‐specific antigen
VEGF: vascular endothelial growth factor

## Supporting information


**DATA S1.** Supporting Information

## Data Availability

The data that supports the findings of this study are available in the supplementary material of this article.
