# Supplementary material for: Cancer vaccines in the clinic
Source: Bioeng Transl Med. 2023 Oct 27;9(1):e10588. doi: 10.1002/btm2.10588 (PMC10771564; doi:10.1002/btm2.10588)
Supplement: Supplementary file 1 — DATA S1. Supporting Information [file BTM2-9-e10588-s001.docx]

**Supporting Information**

**Cancer Vaccines in the Clinic**

Morgan E. Janes,^1,2,3^ Alexander P. Gottlieb,^1,2^ Kyung Soo Park,^1,2^ Zongmin Zhao,^4,5^ and Samir Mitragotri^1,2,†^

^1^ John A. Paulson School of Engineering & Applied Sciences, Harvard University, Cambridge, MA 02138, USA

^2^ Wyss Institute for Biologically Inspired Engineering, Boston, MA 02115, USA

^3^ Harvard-MIT Division of Health Sciences and Technology, Massachusetts Institute of Technology, Cambridge, MA 02139, USA

^4^Department of Pharmaceutical Sciences, College of Pharmacy, University of Illinois Chicago, Chicago, IL 60612, USA

^5^University of Illinois Cancer Center, Chicago, IL 60612, USA

^†^Corresponding author

Email: [mitragotri@seas.harvard.edu](mailto:mitragotri@seas.harvard.edu)

**Supplementary Table 1.** Indications for peptide vaccine clinical trials.

| Indication | Mentions | % of Trials |
| --- | --- | --- |
| Brain | 24 | 14.9% |
| Lung | 19 | 11.8% |
| Breast | 17 | 10.6% |
| Skin | 13 | 8.1% |
| Ovary | 10 | 6.2% |
| Colorectal | 9 | 5.6% |
| Blood | 8 | 5.0% |
| Pancreas | 8 | 5.0% |
| Head and neck | 8 | 5.0% |
| Gastric | 7 | 4.3% |
| Prostate | 5 | 3.1% |
| Cervix | 5 | 3.1% |
| Urothelial and bladder | 5 | 3.1% |
| Other | 23 | 14.3% |

**Supplementary Table 2.** Peptide antigen identities.

| Antigen | Mentions |
| --- | --- |
| hTERT | 11 |
| HER2 | 9 |
| Survivin | 9 |
| WT1 | 5 |
| HPV E6/E7 | 4 |
| NY-ESO-1 | 4 |
| gp100 | 3 |
| UCP2 | 3 |
| CMV pp65 | 3 |
| H3.3K27M | 3 |
| MUC1 | 2 |
| Cancer testis antigens | 2 |
| Carbohydrate antigen | 2 |
| IDH1 R132H | 2 |
| CEA | 2 |
| MAGE-A2 | 2 |
| MAGE-A3 | 2 |
| p53 | 2 |
| PADRE | 2 |
| KRAS | 2 |
| PD-L1 | 2 |
| IDO | 2 |
| GD2L | 2 |
| GD3L | 2 |
| XBP1 | 2 |
| CS1 | 2 |
| RhoC | 1 |
| DNAJB1-PRKACA fusion kinase | 1 |
| CD138 | 1 |
| HSP90 | 1 |
| CD38 | 1 |
| EPHA2 | 1 |
| Labyrinthin | 1 |
| Folate receptor alpha | 1 |
| FOXM1 | 1 |
| DEPDC1 | 1 |
| KIF20A | 1 |
| URLC10 | 1 |
| VEGFR1 | 1 |
| Glioblastoma antigens | 1 |
| BCAN | 1 |
| CSPG4 | 1 |
| FABP7 | 1 |
| IGF2BP3 | 1 |
| NLGN4x | 1 |
| NRCAM | 1 |
| PTPRZ1 | 1 |
| TNC | 1 |
| MET | 1 |
| Hepatitis B virus core antigen | 1 |
| Mutant calreticulin | 1 |
| GAA | 1 |
| Cytomegalovirus gB | 1 |
| Tyrosinase | 1 |
| MAGE-A1 | 1 |
| MAGE-A10 | 1 |
| Vimentin | 1 |
| Alpha-enolase | 1 |
| Estrogen receptor | 1 |

**Supplementary Table 3.** Indications for RNA vaccine clinical trials.

| Indication | Mentions | % of Trials |
| --- | --- | --- |
| Skin | 4 | 15.4 |
| Anogenital | 3 | 11.5 |
| Head and neck | 2 | 7.7 |
| Female reproductive | 2 | 7.7 |
| Esophagus | 2 | 7.7 |
| Lung | 2 | 7.7 |
| Gastric | 2 | 7.7 |
| Other | 9 | 34.6 |

**Supplementary Table 4.** Indications for DNA vaccine clinical trials.

| Indication | Mentions | % of Trials |
| --- | --- | --- |
| Breast | 7 | 18.9 |
| Anogenital | 5 | 13.5 |
| Prostate | 4 | 10.8 |
| Lung | 4 | 10.8 |
| Brain | 2 | 5.4 |
| Skin | 2 | 5.4 |
| Kidney | 2 | 5.4 |
| Urothelial/bladder | 2 | 5.4 |
| Head and neck | 2 | 5.4 |
| Cervix | 2 | 5.4 |
| Multiple/other | 5 | 13.5 |

**Supplementary Table 5.** Indications for viral/heterologous vaccine clinical trials.

| Indication | Mentions | % of Trials |
| --- | --- | --- |
| Prostate | 9 | 12.9 |
| Colorectal | 8 | 11.4 |
| Gastric/GEJ/bowel | 8 | 11.4 |
| Lung | 6 | 8.6 |
| Anogenital | 5 | 7.1 |
| Breast | 5 | 7.1 |
| Pancreas | 5 | 7.1 |
| Head and neck | 4 | 5.7 |
| Blood | 2 | 2.9 |
| Skin | 2 | 2.9 |
| Multiple/other | 16 | 22.9 |

**Supplementary Table 6.** Indications for DC/APC vaccine clinical trials.

| Indication | Mentions | % of Trials |
| --- | --- | --- |
| Brain | 30 | 23.6 |
| Blood | 15 | 11.8 |
| Lung | 10 | 7.9 |
| Skin | 10 | 7.9 |
| Anogenital | 8 | 6.3 |
| Breast | 8 | 6.3 |
| Colorectal | 6 | 4.7 |
| Ovary | 6 | 4.7 |
| Liver | 4 | 3.1 |
| Pancreas | 3 | 2.4 |
| Kidney | 3 | 2.4 |
| Fallopian tube | 3 | 2.4 |
| Peritoneum | 3 | 2.4 |
| Head and neck | 3 | 2.4 |
| Eye | 2 | 1.6 |
| Cervix | 2 | 1.6 |
| Other | 6 | 4.7 |
| Multiple/unknown | 5 | 3.9 |

**Supplementary Table 7.** DC/APC antigen identities.

| Antigen | Mentions |
| --- | --- |
| WT1 | 9 |
| HER2 | 6 |
| Cytomegalovirus pp65 | 5 |
| Survivin | 5 |
| hTERT | 4 |
| NY-ESO-1 | 4 |
| MART-1 | 3 |
| Tumor blood vessel antigens | 2 |
| MAGE-A3 | 2 |
| HPV16 (E6/E7) | 2 |
| MUC1 | 2 |
| Stem-like cells associated antigens | 1 |
| Folate receptor alpha | 1 |
| DKK1 | 1 |
| gp100 | 1 |
| Tyrosinase | 1 |
| PRAME | 1 |
| IDO | 1 |
| Glioblastoma stem cell-like antigens | 1 |
| TRP2 | 1 |
| KRAS | 1 |
| p53 | 1 |
| HER3 | 1 |
| CEA | 1 |
| MAGE-A4 | 1 |
| Multi-MAGE | 1 |
| MG-7 | 1 |
| BCR-ABL | 1 |
| Proteinase-3 | 1 |

**Supplementary Table 8.** Tumor cell genetic modifications.

| Genes | Trials |
| --- | --- |
| GM-CSF | 13 |
| OX40L Ig, gp96 | 1 |
| gp96 Ig | 1 |
| IL-2, XCL1 | 1 |
| GM-CSF, CD40L | 1 |
| IL-2, lymphotactin | 1 |
| GM-CSF, TGF-β, furin knockdown | 1 |

**Supplementary Table 9.** Indications for tumor cell vaccine clinical trials.

| Indication | Mentions | % of Trials |
| --- | --- | --- |
| Pancreas | 8 | 38.1 |
| Brain | 3 | 14.3 |
| Blood | 2 | 9.5 |
| Colorectal | 1 | 4.8 |
| Liver | 1 | 4.8 |
| Skin | 1 | 4.8 |
| Lung | 1 | 4.8 |
| Head and neck | 1 | 4.8 |
| Ovary | 1 | 4.8 |
| Multiple | 2 | 9.5 |
